# Hairline as a Neurosurgical Landmark

**DOI:** 10.7759/cureus.107582

**Published:** 2026-04-23

**Authors:** Oleg Titov, Igor Pronin

**Affiliations:** 1 Department of Neurosurgery, Moscow Multidisciplinary Clinical Center "Kommunarka", Moscow, RUS; 2 Department of Radiology, N.N. Burdenko National Medical Research Center for Neurosurgery, Moscow, RUS

**Keywords:** anatomic landmarks, craniometric points, hairline, neuroanatomy, neuroimaging, neuronavigation, neurosurgery, trichology

## Abstract

Hair is the first layer of the head. It is known from trichology that the hairline forms relatively constant patterns. However, its relation to the deeper structures has remained unexplored. Our goal was to analyze hairline topography with respect to the neuroanatomical structures, as well as to determine the accuracy and representativeness of the identified landmarks.

A prospective study of two sequential groups of patients was conducted. The first group included 500 patients, in whom the general hairline structure and the level of its constancy were determined, depending on sex. The second group included 20 patients (40 head sides). In these patients, a radiopaque wire was fixed along the hairline, CT angiography (CTA) was performed and combined with incoming MRI, and finally, detailed topographic analysis was performed using 3D visualization.

For descriptive purposes, we have developed a new anatomical terminology that divides the hairline into easily distinguishable points and segments. Using these landmarks, we found a clear relationship between the hairline and more than 20 cranial, cerebral, and vascular structures. Specifically, the hairline allows localization of the coronal and lambdoid sutures, sphenoidal and petrosal ridges, C2 vertebra, cortical sulci (Sylvian, central, precentral, superior, and inferior frontal), gyri (precentral, postcentral, all major frontal and temporal), and vessels (superficial temporal artery (STA), sinodural angle, and transverse-sigmoid sinus junction (TSSJ)). Discovered landmarks showed clinically acceptable spatial accuracy (overall shift 6.9±3.3 mm) and representativeness. The frontal region was constant in 85% of males (n=211) and 99% of females (n=249), while the temporal and occipital regions were from 95% (n=238) to 100% (n=250) of males and females.

In conclusion, the hairline is topographically connected with deep layers. The novel data can be used to plan neurosurgical approaches, in addition to standard craniometric landmarks and neuronavigation.

## Introduction

Anatomical orientation in the cranial space is a crucial skill for a neurosurgeon. Modern technologies of neuronavigation allow the effective digital visualization of the surgical target and trajectory [1,2]. However, clinical navigation based on real anatomical landmarks remains all-important, especially since the navigation stations are still not present in some hospitals and may be subject to technical failures [3,4].

In craniocerebral anatomy, a lot of landmarks on the skin, skull, and cerebral cortex are known: nasion, tragus, skull sutures, sulcal points, etc., which are widely used in planning neurosurgical approaches [5-9]. However, there is another layer that has been studied much less, although it is located right under the nose-the hair. According to trichology, the hairline forms relatively constant, typical patterns [10-13]. The question is, how does it relate to the deep structures? Could it be useful for neurosurgeons?

Our goal was to analyze the hairline patterns and their topography in relation to surgically significant neuroanatomic structures, as well as to determine the accuracy and representativeness of the identified landmarks.

## Materials and methods

Study design

A single-center prospective study was performed in the Burdenko Neurosurgery Center, Moscow, Russia, in 2024-2025. The local Institutional Ethics Committee approved the study (approval number: 2023-12). All patients signed an informed consent form.

Two groups of neurosurgical patients were sequentially recruited (Appendix A).

The first group included 500 adults (250 males and 250 females), aged 18 to 84 years (median 49 years), passing through the admissions unit for routing to specialized neurosurgical departments. Four hundred and seventy-eight (95.6%) patients were Caucasian, and 22 (4.4%) were Asian. This group was necessary for a visual analysis of the general hairline structure, determining its consistency, and evaluating the representativeness of the found landmarks.

The second group included 20 Caucasian adults (10 males and 10 females), aged 18 to 82 years (median 47 years), hospitalized for elective neurosurgical treatment, according to the criteria: intact hairline, soft tissues, cranial vault, and convexital surface of the cerebral cortex; high-quality incoming MRI; and possibility of performing CT angiography (CTA) before surgery. This group was necessary for a detailed neuroimaging-based analysis of the hairline and its relation to the neuroanatomical structures.

Sample size estimation

This is a first-in-human pilot study, which is not based on existing data, so the usual statistical power calculation was not applicable. As discussed elsewhere, for pilot studies with continuous outcomes, a sample size of 12 to 35 per group is needed [14]. In some classical works on neurosurgical craniometry, where one side of the head (face, brain, skull, etc.) is considered as one sample, 10 to 32 samples have been used [5,6,9,15]. In a recent publication in the official journal of the American and British Associations of Clinical Anatomists, a minimal standard for modern anatomical research has been proposed: at least 10 samples [16]. As for trichology, as a rule, hundreds of patients are examined (from 469 to 3897 [10-12, 17-19]), since external head examination is relatively easy to perform.

Given the above reasons, we decided to examine 500 patients in the first stage and 20 patients (i.e., 40 head sides) in the second stage, wherein all the hairlines represented the average pattern from the first group.

Data collection

In both groups, the hairline consistency was assessed using the standard trichological classification, Basic and Specific (BASP) [10]. This is a visual analog tool that allows one to assess the severity of androgenic alopecia. In the BASP classification, hairline patterns are designated according to Latin letters, resembling the atrophied hairline, M, C, U, etc. L means "linear". Digital indices indicate the degree of atrophy. In the research process, the BASP classification was slightly modified (Appendix B). The L, М0, and С0 patterns were combined into the L pattern since they essentially did not differ. Patterns L, M1, and С1 were considered the norm of the frontal region; patterns М2, М3, С2, С3, U1, U2, and U3 were considered its atrophy. The orbital and zygomatic triangles of the temporal region were classified as normal (if present) or atrophied (if absent). The occipital region was recognized as normal when located at the level of the craniovertebral junction (depression between the occiput and neck) and atrophied when located higher, at the level of the occipital bone. Total alopecia was encoded as the O pattern.

In the second group, the patients underwent preoperative CTA on the GE Optima CT660 CT scanner (GE Healthcare, Chicago, IL, USA) according to the protocol: all structures above the C5 vertebra, native, arterial, and venous phases; and axial sections 0.625 mm thick. Before the examination, a radiopaque copper wire was fixed along the hairline using a surgical patch.

In the Inobitec PRO program (version 2.15.2, Inobitec Software, Voronezh, Russia), MRI and CTA were fused and segmented into the hairline, skin, skull, brain, and blood vessels (Appendix C). In the resulting images, the hairline segments were measured, and their topographic analysis was performed.

Topographic analysis

Initially, the hairline topography was evaluated qualitatively by the proximity of its key points and segments to the clinically significant neuroanatomical structures: sutures, sulci, etc. Next, the topography was quantified (Appendix D).

In each case, two points were identified, which were the landmark and the target. The shift was measured, the distance between these points in mm. The direction of shift was determined by four vectors: medial-lateral, anterior-posterior, anteromedial-posterolateral, and anterolateral-posteromedial; the landmark point was considered 0. When the target point was located in the positive vector part, the shift was recorded with a plus, and vice versa. The average position of the target point on these axes was calculated. This gave a correction reflecting where to move relative to the landmark in order to get to the target point with the least shift.

Each case was remodeled, given the shifts and corrections; the latter were considered effective in case of a clinically significant increase in accuracy.

The landmarks' accuracy was estimated by the resulting average shifts (taking into account effective corrections), which were additionally rounded to intervals of 5 mm according to the nearest neighbor principle and presented as categories: A (shift tends to 0 mm), B (shift tends to 5 mm), C (shift tends to 10 mm), and D (shift tends to 15 mm).

If the landmark was a point, and the target structure was linear, then the shift was determined as the shortest distance between them, and only one necessary vector was used (e.g., anterior-posterior). If both structures were linear, then the shortest distances between their ends and midpoints were determined; these three distances were averaged, and thus a shift was obtained.

Statistics

Statistics were performed in the Jamovi software (version 2.3.28; jamovi project, Sydney, Australia). Depending on the task, the following methods were used: descriptive statistics, paired T-test for independent samples (Student and Mann-Whitney), paired T-test for dependent samples (Student and Wilcoxon), Fisher's exact test. The statistical significance was set at p<0.05.

Visualization

All illustrations were created by the first author (OT) using Inobitec PRO software and Microsoft PowerPoint 2013 (version 15.0.5579.1001; Microsoft Corporation, Redmond, WA, United States).

## Results

Hairline structure

**Figure 1:**
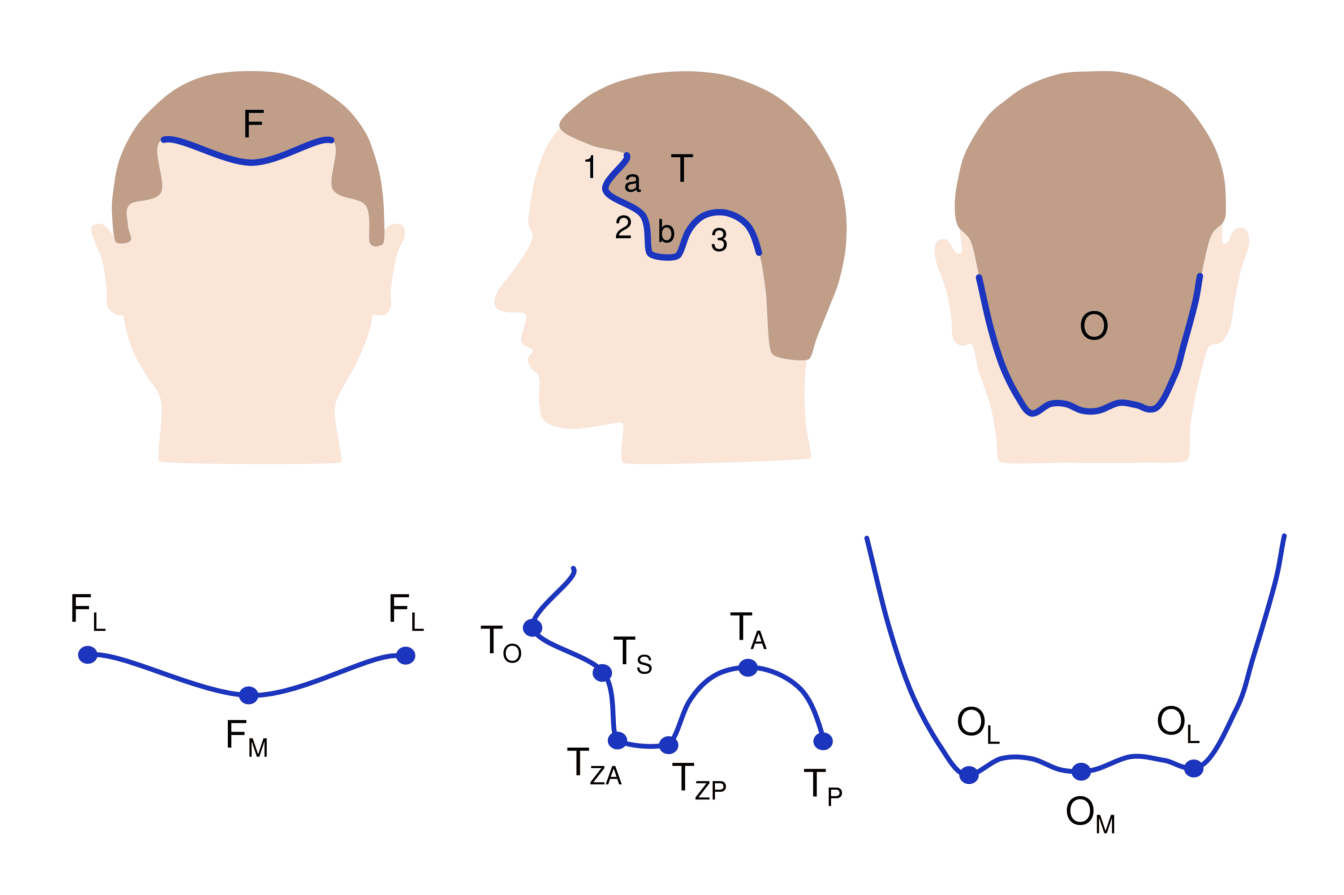
General hairline structure Upper row: frontal (F), temporal (T), and occipital (O) regions. Lower row: key points and segments. a: orbital triangle; b: zygomatic triangle; 1: fronto-temporal angle; 2: sphenoidal angle; 3: auricular arc. This figure has been created by the authors using Microsoft PowerPoint (Microsoft Corporation, Redmond, WA, United States).

To find out the hairline anatomy, we observed 500 adult neurosurgical patients (first group), and developed a simple universal scheme, which divides the hairline into three regions (frontal, temporal, and occipital), 18 points, and 18 segments (Figure 1)

The frontal region is unpaired, has a V-shape, and includes three key points. Median frontal (F_M_) is the unpaired intersection of the frontal hairline and the median line. Lateral frontal (F_L_) is the paired apex of the frontal-temporal angle, which represents the junction of the frontal and temporal hairline.

The temporal region is paired, W-shaped, and contains two “triangles”; the first is directed at the eye and therefore is called “orbital”, the second is directed at the zygomatic arch and is called “zygomatic”. Between them, there is a sphenoidal angle. In males, the zygomatic triangle may turn into a beard, in which case the upper edge of the zygomatic arch is considered its lower boundary. The posterior part of the temporal region surrounds the ear and forms a C-shaped “auricular arc”. This region includes six points. Orbital (T_O_) is the apex of the orbital triangle. Sphenoidal (T_S_) is the apex of the sphenoidal angle. Anterior zygomatic (T_ZA_) is the anterior lower edge of the zygomatic triangle. Posterior zygomatic (T_ZP_) is the posterior lower edge of the zygomatic triangle. Auricular (T_A_) is the top of the auricular arc, above the external auditory canal. Petrosal (T_P_) is the intersection of the upper edge of the zygomatic root (ZR) with the posterior half of the auricular arc; it separates the temporal and occipital regions.

The occipital region is unpaired, has a U (sometimes bat tail) shape, and includes three points. Median occipital (O_M_) is unpaired, located on the neck, at the intersection of the midline with the occipital hairline. Lateral occipital (O_L_) is paired, located at the posterior edge of the sternocleidomastoid muscle.

Between the hair points, the corresponding segments are located (F_M_-F_L,_ etc.).

Hairline consistency

Given that neurosurgeons sometimes encounter “bald” patients, we examined the occurrence of loss of certain hairline parts. Among the 500 patients (250 males, 250 females), the hairline integrity significantly depended on sex (Appendix E). Males more frequently had the atrophy of the frontal and occipital regions than females; the temporal regions were comparable.

In males (n=250), intact patterns were found with the following frequency: frontal region, 84.4% (n=211); orbital and zygomatic triangles of the temporal region, 95.2% (n=238) and 99.2% (n=248); occipital region, 97.2% (n=243). 

In the absolute majority of females (n=250), all the hairline parts were intact: frontal region, 99.6% (n=249); orbital and zygomatic triangles, 97.2% (n=243) and 100% (n=250); occipital region, 100% (n=250).

Virtual hairline analysis

Further, for a targeted study of the topographic hairline anatomy, we recruited 20 more neurosurgical patients, the second group, 10 males and 10 females (40 head sides in total), reproducing the norm from the first group. The male hairline patterns did not differ from the female ones (p=0.489); thus, the sample was homogeneous in the main parameter.

In these patients, we fixed a radiopaque wire along the hairline using a medical patch and performed CTA of the head. The CTA was merged with the incoming MRI. The resulting images were analyzed using 3D visualization.

We measured all the hairline parts (Table 1). The total length of the hairline was 79±4 (71.5-88.5) cm. The frontal region in males was on average 3 cm longer (males: 15.5±1.5 (13.5-18.0) cm, females: 12.5±2.0 (9.5-14.5) cm). The temporal region on each side was 18.5±1.5 (15.0-23.0) cm, without sex difference. Occipital region was 28.5±2.5 (24.0-34.0) cm, without sex difference. There was no left-right difference in any segment.

**Table 1:** Length of the hairline parts M: male; F: female; L: left; R: right; N/A: not applicable; F_M_: median frontal point; F_L_: lateral frontal point; T_O_: orbital point; T_S_: sphenoidal point; T_ZA_: anterior zygomatic point; T_ZP_: posterior zygomatic point; T_A_: auricular point; T_P_: petrosal point; O_L_: lateral occipital point; O_M_: median occipital point; *statistically significant differences (p<0.05).

Hairline part	Length, cm					M vs. F	L vs. R
-	Average	Male	Female	L	R	-	-
Frontal region	14.0±2.0 (9.5–18.0)	15.5±1.5 (13.5–18.0)	12.5±2. (9.5–14.5)	N/A		p<0.001*	N/A
F_M_ – F_L_	7.0±1.0 (4.0–10.0)	8.0±1.0 (6.5–10.0)	6.0±1.0 (4.0–8.0)	7.0±1.0 (5.5–10.0)	6.5±1.0 (4.0–8.5)	p<0.001*	p=0.155
Temporal region	18.5±1.5 (15.0–23.0)	18.5±1.0 (16.5–20.5)	18.0±2.0 (15.0–23.0)	18.0±2.0 (15.0–23.0)	18.5±1.5 (15.0–21.0)	p=0.214	p=0.482
F_L_ – T_O_	4.0±1.0 (2.5–7.0)	4.5±1.0 (2.5–7.0)	3.5±1.0 (2.5–5.5)	4.0±1.0 (2.5–5.5)	4.5±1.0 (2.5–7.0)	p<0.001*	p=0.232
T_O_ – T_S_	2.5±0.5 (2,0–3,5)	2.5±0.5 (2.0–3.0)	2.5±0.5 (2.0–3.5)	2.5±0.5 (2.0–3.0)	3.0±0.5 (2.0–3.5)	p=0.405	p=0.506
T_S_ – T_ZA_	2.5±0.5 (2.0–3.5)	2.5±0.5 (2.0–3.5)	2.5±0.5 (2.0–3.5)	2.5±0.5 (2.0–3.5)	2.5±0.5 (2.0–3.5)	p=0.211	p=0.131
T_ZA_ – T_ZP_	2.0±0.5 (1.0–3.0)	2.0±0.5 (1.0–3.0)	1.5±0.5 (1.0–2.5)	2.0±0.5 (1.0–2.5)	2.0±0.5 (1.0–3.0)	p=0.14	p=0.645
T_ZP_ – T_A_	3.5±1.0 (2.5–6.5)	3.5±0.5 (3.0–4.5)	4.0±1.0 (2.5–6.5)	4.0±1.0 (3.0–6.5)	3.5±0.5 (2.5–5.0)	p=0.871	p=1.0
T_A_ – T_P_	3.5±0.5 (2.5–4.5)	3.5±0.5 (2.5–4.5)	3.5±0.5 (2.5–4.5)	3.5±0.5 (2.5–4.5)	3.5±0.5 (2.5–4.5)	p=0.421	p=0.17
Occipital region	28.5±2.5 (24.0–34.0)	28.0±3.0 (24.0–34.0)	29.5±2.5 (25.5–34.0)	N/A		p=0.104	N/A
T_P_ – O_L_	8.5±1.5 (4.5–11.0)	8.0±1.5 (4.5–11.0)	9.0±1.0 (7.5–10.5)	8.5±1.5 (4.5–10.5)	8.5±1.5 (5.5–11.0)	p=0.02*	p=0.741
O_L_ – O_M_	6.0±1.5 (4.0–10.5)	6.0±1.5 (4.5–8.5)	5.5±1.5 (4.0–10.5)	6.0±1.5 (4.5–10.5)	5.5±1.5 (4.0–8.5)	p=0.291	p=0.456
Total hairline	79.0±4.0 (71.5–88.5)	80.5±2.0 (77.5–85.5)	77.5±5.0 (71.5–88.5)	39.5±2.5 (36.5–47.0)	39.5±2.0 (34.5–42.5)	p=0.015*	p=0.64

**Figure 2:**
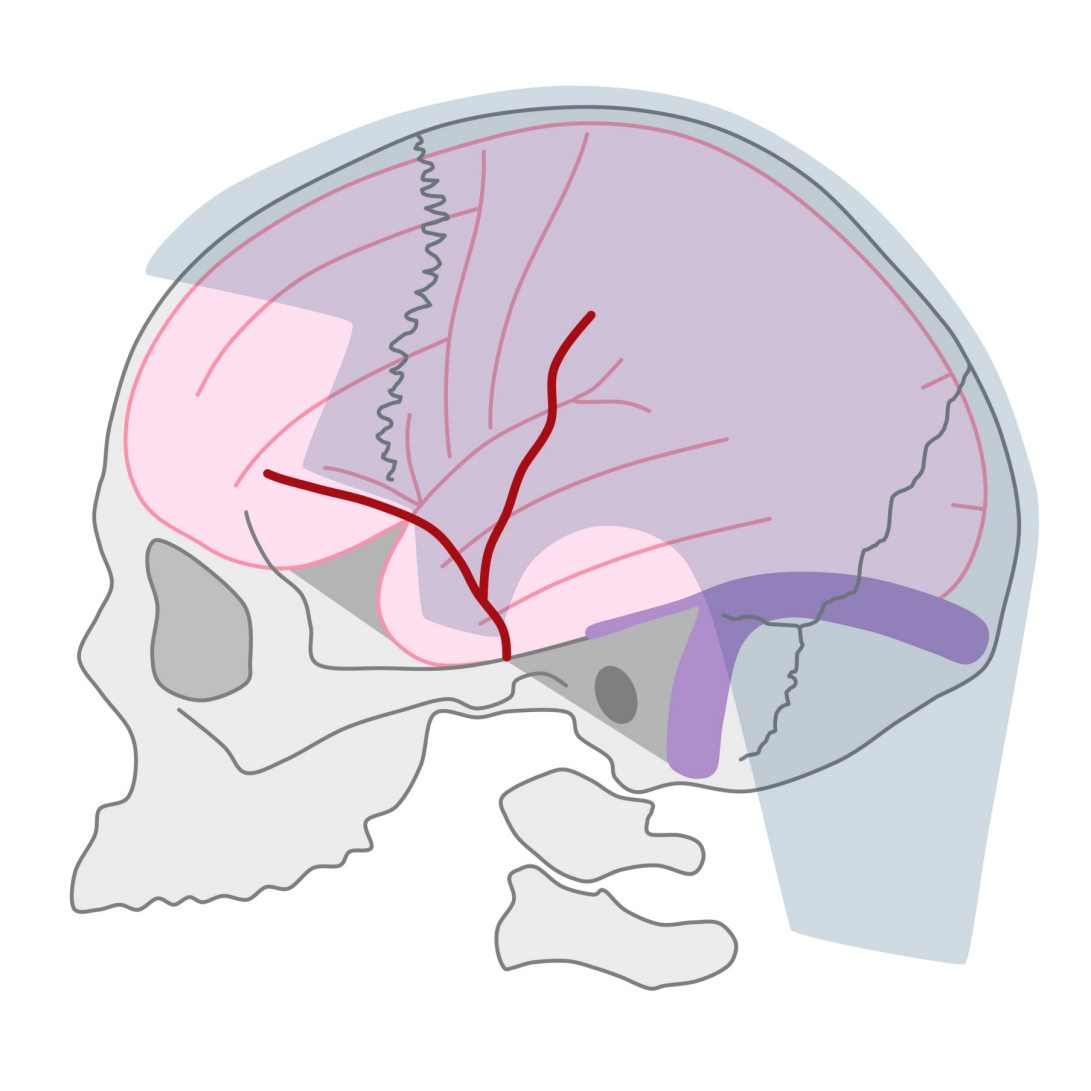
Hairline relation to neuroanatomical structures: the general picture Hair zone is shaded. Grey color: skull bones and vertebrae (note the sphenoidal and petrosal ridges); Pink color: brain cortex; Red color: superficial temporal artery; Purple color: transverse, sigmoid, and superior petrosal sinuses. This figure has been created by the authors using Microsoft PowerPoint (Microsoft Corporation, Redmond, WA, United States).

Topographic analysis revealed the hairline relationship with more than 20 neuroanatomical structures (Figure 2). Despite some individual variability, the overall picture was fairly stable. Below, there is a text description of the average topographic hairline portrait. Appendices F-G include the full qualitative and quantitative topographic data.

Frontal hairline topography

The F_M_ Point

In all cases, the F_M_ point was located between nasion and bregma. The nasion-F_M_ and F_M_-bregma segments were 6.5 cm long (shift 8.9±6.1 and 8.1±5.9 mm respectively, category C), with no significant difference (p=0.841). 

**Figure 3:**
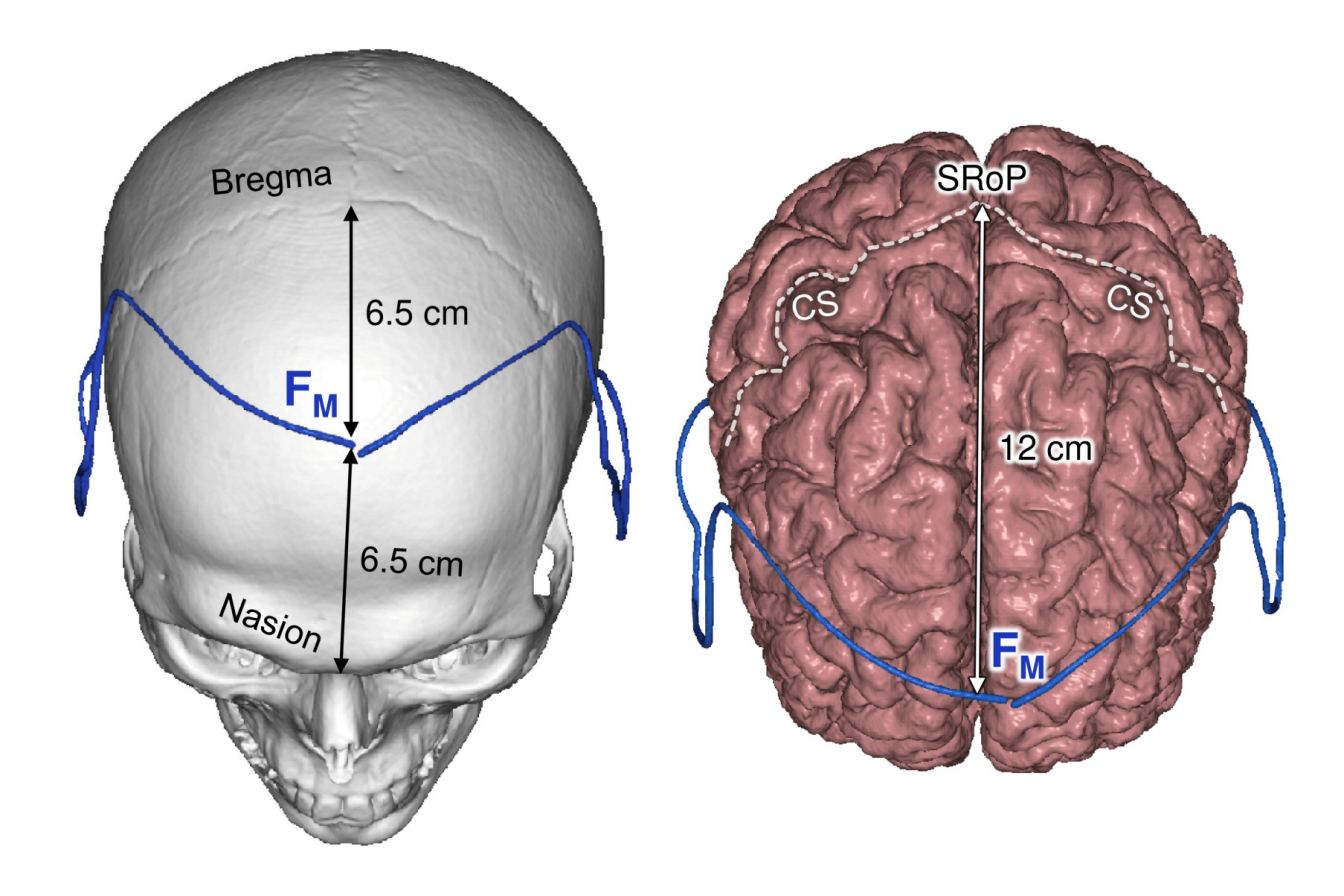
Median frontal point topography Left: projection on the skull bones; Right: projection on the cerebral cortex; F_M_: median frontal point; CS: central sulcus; SRoP: superior rolandic point. This figure has been created by the authors using Inobitec PRO software (version 2.15.2; Inobitec Software, Voronezh, Russia) and Microsoft PowerPoint (Microsoft Corporation, Redmond, WA, United States).

The average distance from the F_M_ point to the superior rolandic point (SRoP) was 12 cm (shift 7.7±5.1 mm, category C). The SRoP is located at the intersection of the central sulcus and the interhemispheric fissure, on average 5 cm posterior to the bregma, and is an important landmark for the localization of the primary motor and sensory cortex (Figure 3) [6,7].

The F_M_-F_L_ Midpoint

**Figure 4:**
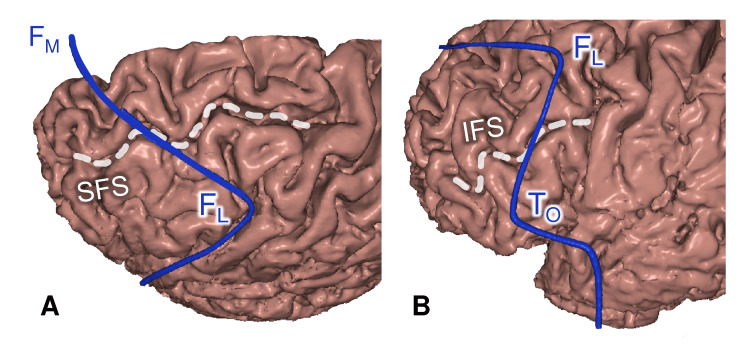
Hairline intersection with the frontal sulci (A) F_M_–F_L_ midpoint and the superior frontal sulcus (SFS). (B) F_L_–T_O_ midpoint and the inferior frontal sulcus (IFS). FL: lateral frontal point; FM: median frontal point; TO: orbital point This figure has been created by the authors using Inobitec PRO software (version 2.15.2; Inobitec Software, Voronezh, Russia) and Microsoft PowerPoint (Microsoft Corporation, Redmond, WA, United States).

In most cases, the F_M_-F_L_ midpoint was projected on the superior frontal sulcus (SFS), where the latter intersects the frontal hairline (shift 3.7±3.4 mm, category B) (Figure 4A).

The F_L_ Point

**Figure 5:**
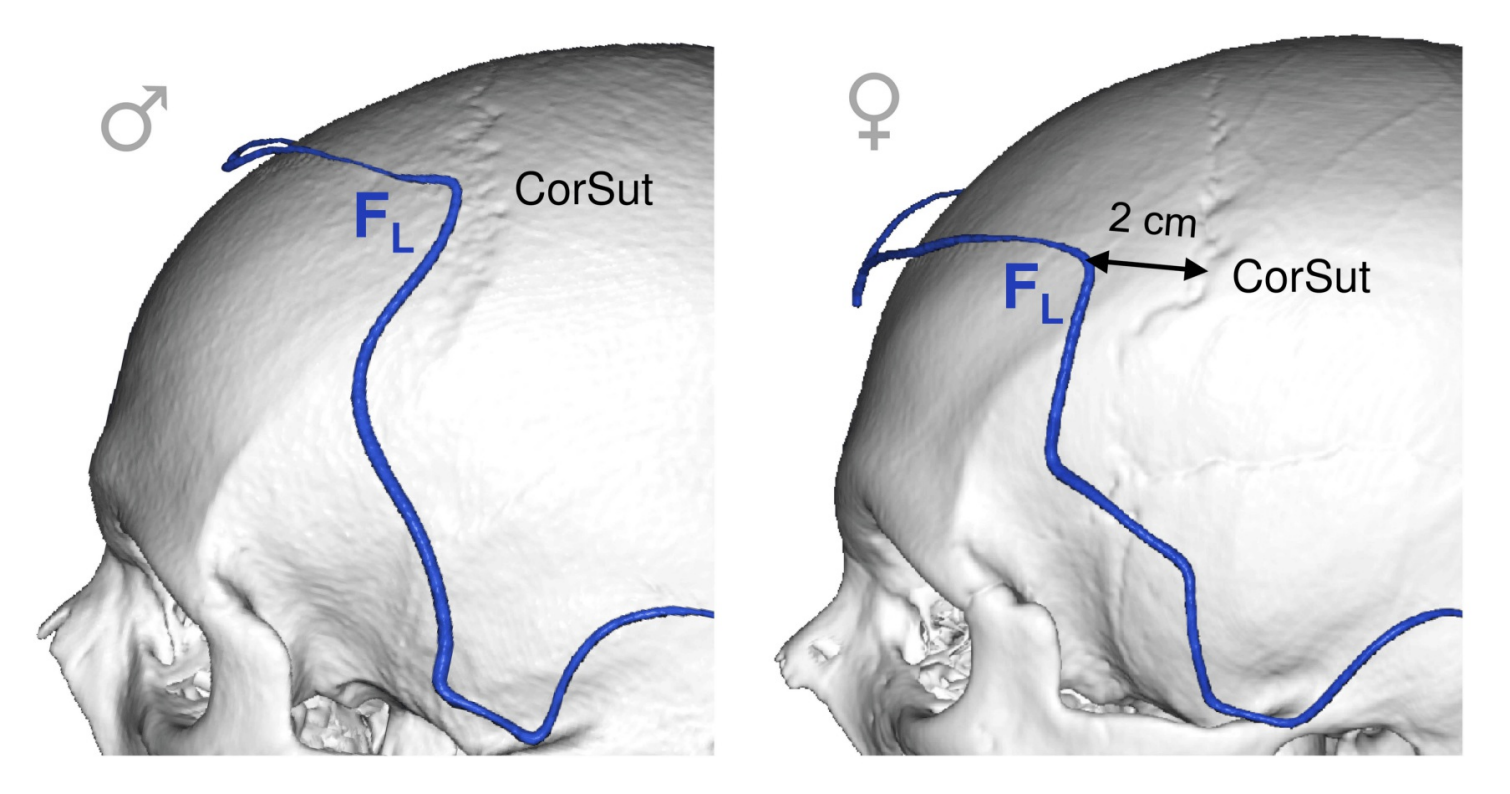
Lateral frontal point topography in relation to the skull bones: males vs. females F_L_: lateral frontal point; CorSut: coronal suture This figure has been created by the authors using Inobitec PRO software (version 2.15.2; Inobitec Software, Voronezh, Russia) and Microsoft PowerPoint (Microsoft Corporation, Redmond, WA, United States).

The F_L_ point was located near the coronal suture: exactly on that in males (shift 8.1±5.7 mm, category C) and 2 cm anterior to it in females (shift 9.2±5.7 mm, category C) (Figure 5). At this point, the hairline is closest to the coronal suture, so the latter is better palpated from the F_L_.

At the brain level, the F_L_ point in males was usually projected on the posterior third of the middle frontal gyrus (MFG), which is the frontal eye field (FEF) [20-22]. In females, it was projected on the middle third of the MFG, which is the dorsolateral prefrontal cortex (DLPFC) [23,24]. 

**Figure 6:**
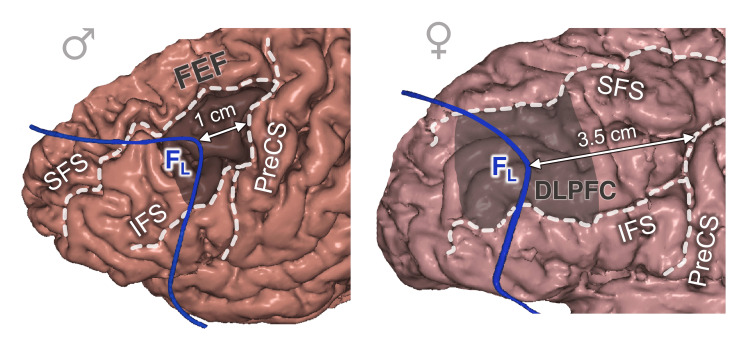
Lateral frontal point topography in relation to the brain cortex, males vs females FL: lateral frontal point; PreCS: precentral sulcus; SFS: superior frontal sulcus; IFS: inferior frontal sulcus; FEF: frontal eye field; DLPFC: dorsolateral prefrontal cortex. This figure has been created by the authors using Inobitec PRO software (version 2.15.2; Inobitec Software, Voronezh, Russia) and Microsoft PowerPoint (Microsoft Corporation, Redmond, WA, United States).

The precentral sulcus was located 1 cm posterior to the F_L_ in males (shift 5.0±6.3 mm, category B) and 3.5 cm posterior to it in females (shift 8.0±7.3 mm, category C) (Figure 6).

Temporal hairline topography

The F_L_-T_O_ Midpoint

In most cases, the F_L_-T_O_ midpoint was projected on the inferior frontal sulcus (IFS), where the latter intersects the frontal hairline (shift 2.2±2.7 mm, category A) (Figure 4B).

The T_O_ Point

**Figure 7:**
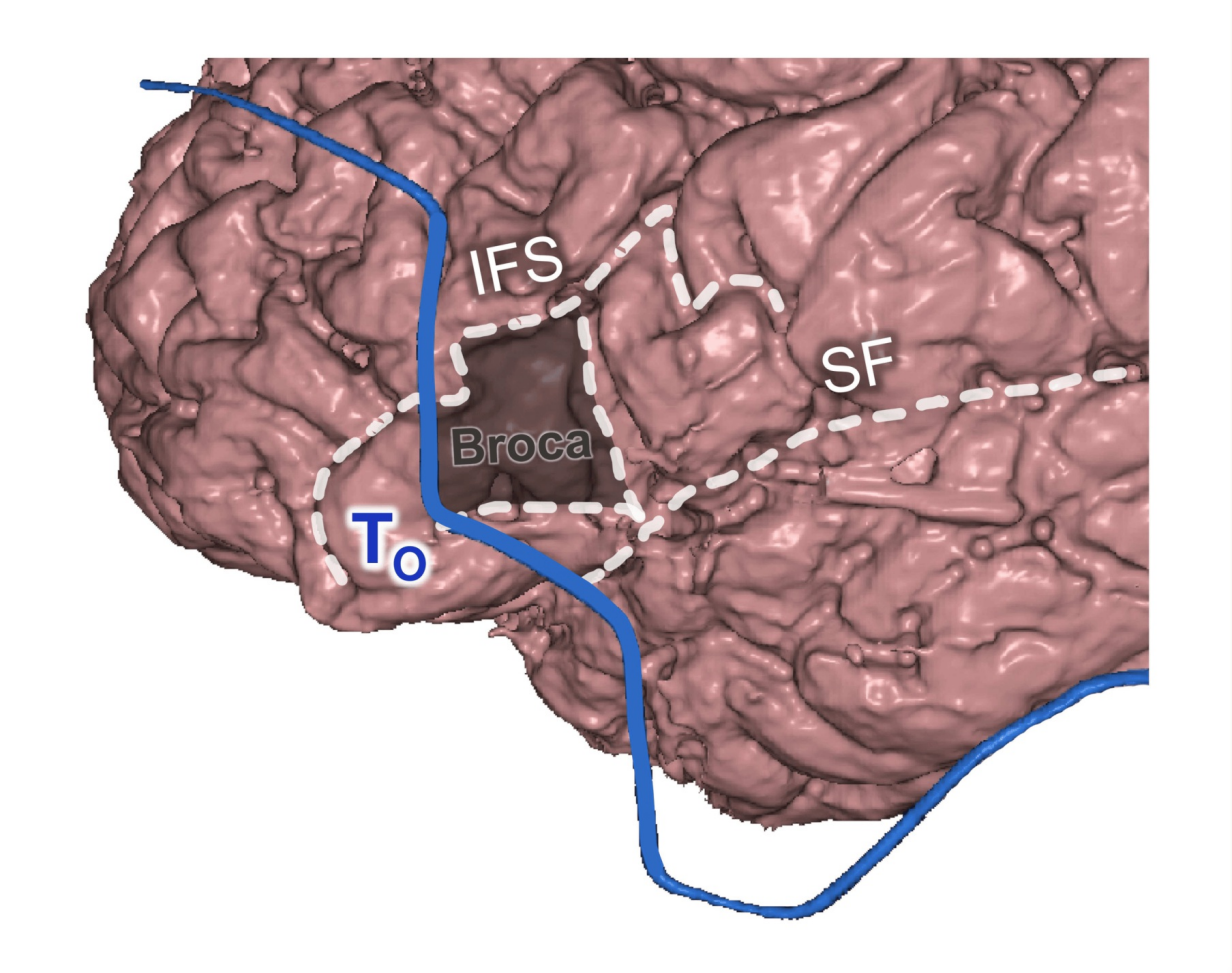
Orbital point topography T_O_: orbital point; SF: Sylvian fissure; IFS: inferior frontal sulcus; Broca: frontal language area This figure has been created by the authors using Inobitec PRO software (version 2.15.2; Inobitec Software, Voronezh, Russia) and Microsoft PowerPoint (Microsoft Corporation, Redmond, WA, United States).

In most cases, the orbital triangle was projected on the posterior third of the inferior frontal gyrus (IFG), which is the Broca language area, or its contralateral counterpart (Figure 7) [25-28].

The T_O_ point was usually projected on the anterior (horizontal) branch of the Ssylvian fissure (SF). The geometric center of the IFG triangular part was located 5 mm posterior to the T_O_ in males (shift 4.9±3.4 mm, category B) and 15 mm posterior to it in females (shift 7.0±3.0 mm, category B).

The T_O_-T_S_ Segment

**Figure 8:**
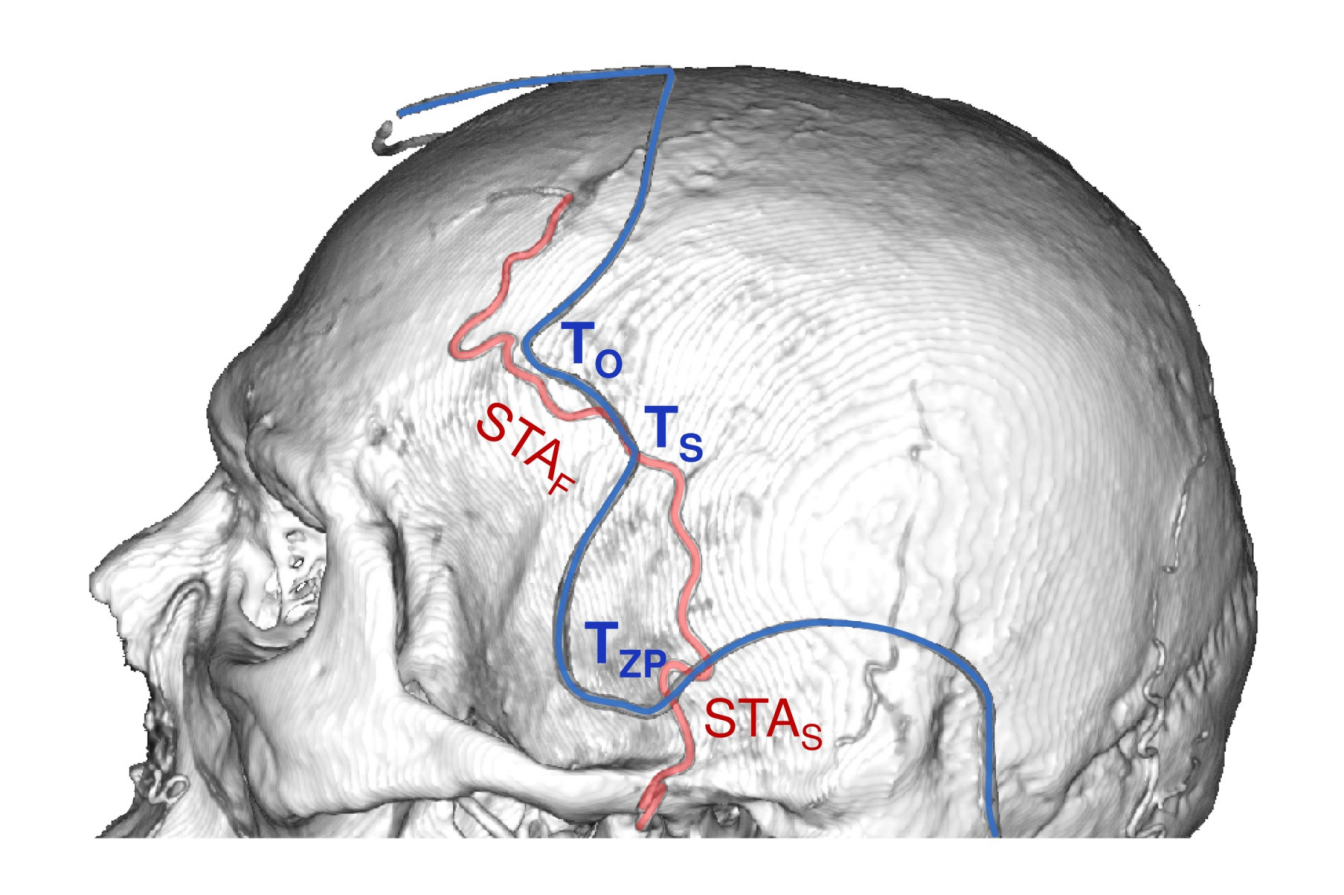
Hairline and the superficial temporal artery T_O_–T_S_ segment coincides with the frontal branch of the superficial temporal artery (STA_F_); T_ZP_ point – with the stem (STA_S_). T_O_: orbital point; T_S_: sphenoidal point; ; T_ZP_: posterior zygomatic point This figure has been created by the authors using Inobitec PRO software (version 2.15.2; Inobitec Software, Voronezh, Russia) and Microsoft PowerPoint (Microsoft Corporation, Redmond, WA, United States).

The T_O_-T_S_ segment pointed to the frontal branch of the superficial temporal artery (STA) (shift 6.7±4.9 mm, category B) (Figure 8).

*The* *T_S_ Point*

The sphenoidal angle was projected on the greater wing of the sphenoid bone and on the sphenoidal ridge, which separates the anterior and middle cranial fossa.

The T_S_ point was usually located on the sphenoidal depression (shift 6.8±4.7 mm, category B) and projected on the lateral end of the sphenoidal ridge - the “T_S_ ridge point” (synonym - sphenopterional point [29]) (shift 10.1±4.9 mm, category C).

**Figure 9:**
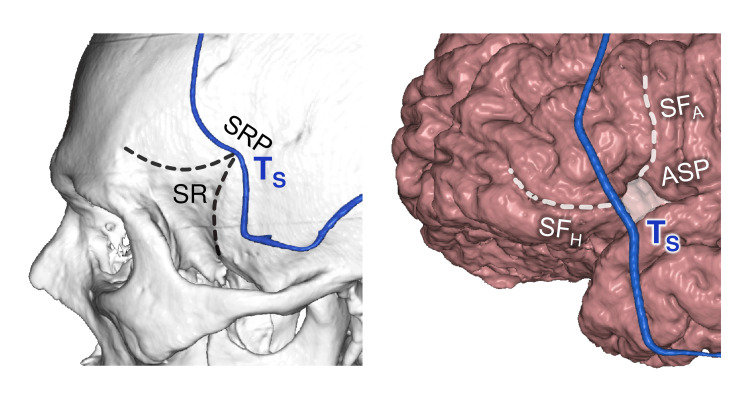
Sphenoidal point topography Left: projection on the skull bones; Right: projection on the cerebral cortex; T_S_: sphenoidal point; SR: sphenoidal ridge; SRP: sphenoidal ridge point; SF_H_ and SF_A_: horizontal and ascending branches of the Sylvian fissure; ASP: anterior Sylvian point. This figure has been created by the authors using Inobitec PRO software (version 2.15.2; Inobitec Software, Voronezh, Russia) and Microsoft PowerPoint (Microsoft Corporation, Redmond, WA, United States).

At the brain level, the T_S_ point was projected on the SF stem (shift 0.3±2.8 mm, category A). The anterior Sylvian point (ASP) was located 5 mm superior to the T_S_ (shift 5.2±3.8 mm, category B) (Figure 9). ASP is located at the convergence of the SF stem, horizontal, and ascending branches, and serves as a starting point for SF dissection [5-7].

The T_ZA_ and T_ZP_ Points

The zygomatic triangle was projected on the anterior third of the temporal lobe.

**Figure 10:**
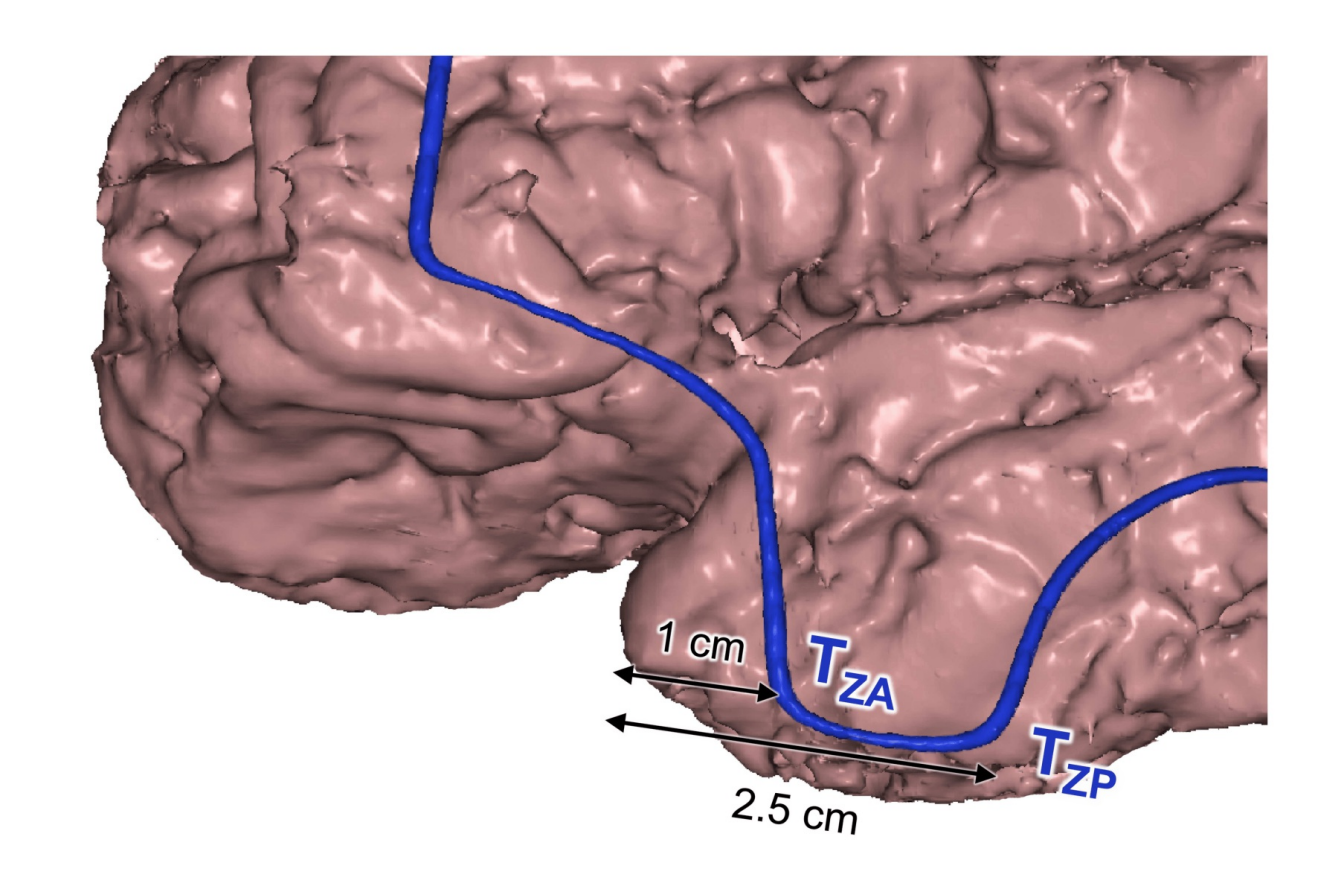
Anterior and posterior zygomatic points topography T_ZA_: anterior zygomatic point; T_ZP_: posterior zygomatic point; Distances to the temporal tip are indicated. This figure has been created by the authors using Inobitec PRO software (version 2.15.2; Inobitec Software, Voronezh, Russia) and Microsoft PowerPoint (Microsoft Corporation, Redmond, WA, United States).

The T_ZA_ point was projected on the temporal pole, 1 cm posterior to its tip (shift 4.1±3.4 mm, category B). T_ZP_ point was most often projected on the inferior temporal gyrus (IFG), 2.5 cm posterior to the temporal tip (shift 3.9±3.1 mm, category B) (Figure 10). T_ZP_ was also projected on the STA stem (shift 3.2±3.0 mm, category B) (Figure 8).

*The* *T_A_ Point*

**Figure 11:**
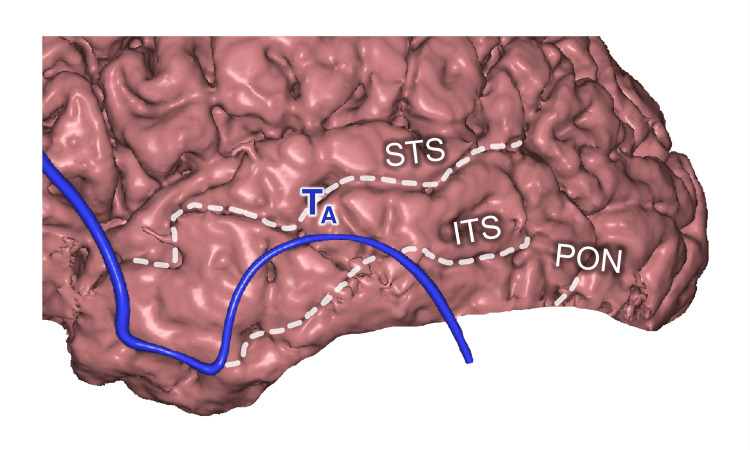
Auricular point topography T_A_: auricular point; STS: superior temporal sulcus; ITS: inferior temporal sulcus; PON: preoccipital notch This figure has been created by the authors using Inobitec PRO software (version 2.15.2; Inobitec Software, Voronezh, Russia) and Microsoft PowerPoint (Microsoft Corporation, Redmond, WA, United States).

The T_A_ point was grossly projected on the middle third of the middle temporal gyrus (MTG) (Figure 11), between the temporal tip and the preoccipital notch (PON).

The T_P_ Point

**Figure 12:**
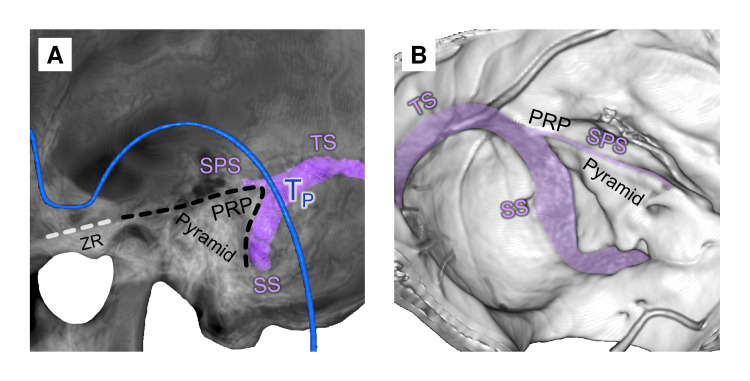
Petrosal point topography (A) Outside skull view (semi-transparent mode); (B) Inside view; T_P_: petrosal point; PRP: petrosal ridge point; TS: transverse sinus; SS: sigmoid sinus; SPS: superior petrosal sinus; ZR: zygomatic root This figure has been created by the authors using Inobitec PRO software (version 2.15.2; Inobitec Software, Voronezh, Russia) and Microsoft PowerPoint (Microsoft Corporation, Redmond, WA, United States).

The base of the auricular arc was projected on the petrous pyramid (Figure 12).

The T_P_ point was projected on the “petrosal ridge point” (PRP) (shift 6.4±4.5 mm, category B). PRP is the lateral end of the petrosal ridge, which separates the middle and posterior cranial fossa and, from the vascular perspective, indicates the position of the sinodural angle, where the superior petrosal sinus runs into the sigmoid sinus [30,31].

The transverse-sigmoid sinus junction (TSSJ) was located 1 cm posterior to the T_P_ (shift 4.1±2.8 mm, category B).

The Supraauricular Line (SAL)

**Figure 13:**
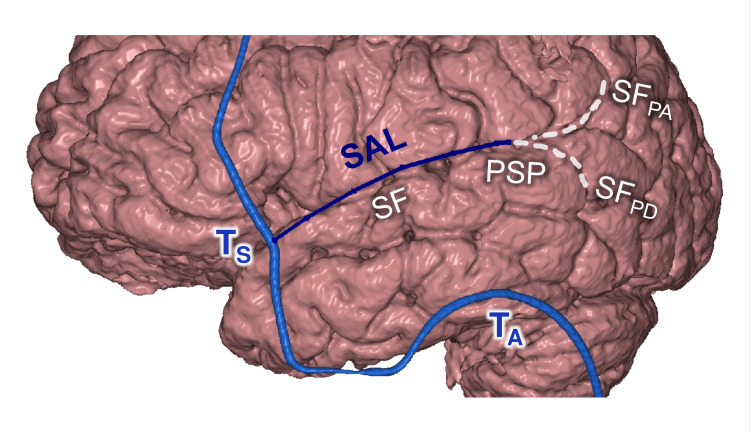
Hairline and the SF T_S_: sphenoidal point; T_A_: auricular point; SAL: supraauricular line; PSP: posterior Sylvian point; SF: Sylvian fissure; SF_PA_ and SF_PD_: posterior ascending and descending SF branches This figure has been created by the authors using Inobitec PRO software (version 2.15.2; Inobitec Software, Voronezh, Russia) and Microsoft PowerPoint (Microsoft Corporation, Redmond, WA, United States).

The SAL is drawn from the T_S_ point parallel to the auricular arc up to the level of the T_A_ point. This line was projected on the SF (shift 3.6±4.7 mm, category B); its posterior end on the posterior sylvian point (PSP) (shift 6.4±5.4 mm, category B) (Figure 13). PSP is the place where the SF divides into posterior ascending and descending branches [5,32]; there are large cortical branches of the middle cerebral artery (MCA), which are used as recipient vessels for brain revascularization [33,34]. Moreover, the Wernicke language area is usually located in the left posterior perisylvian cortex, surrounding the PSP [35,36].

Intersection of SAL and F_L_-T_A_

**Figure 14:**
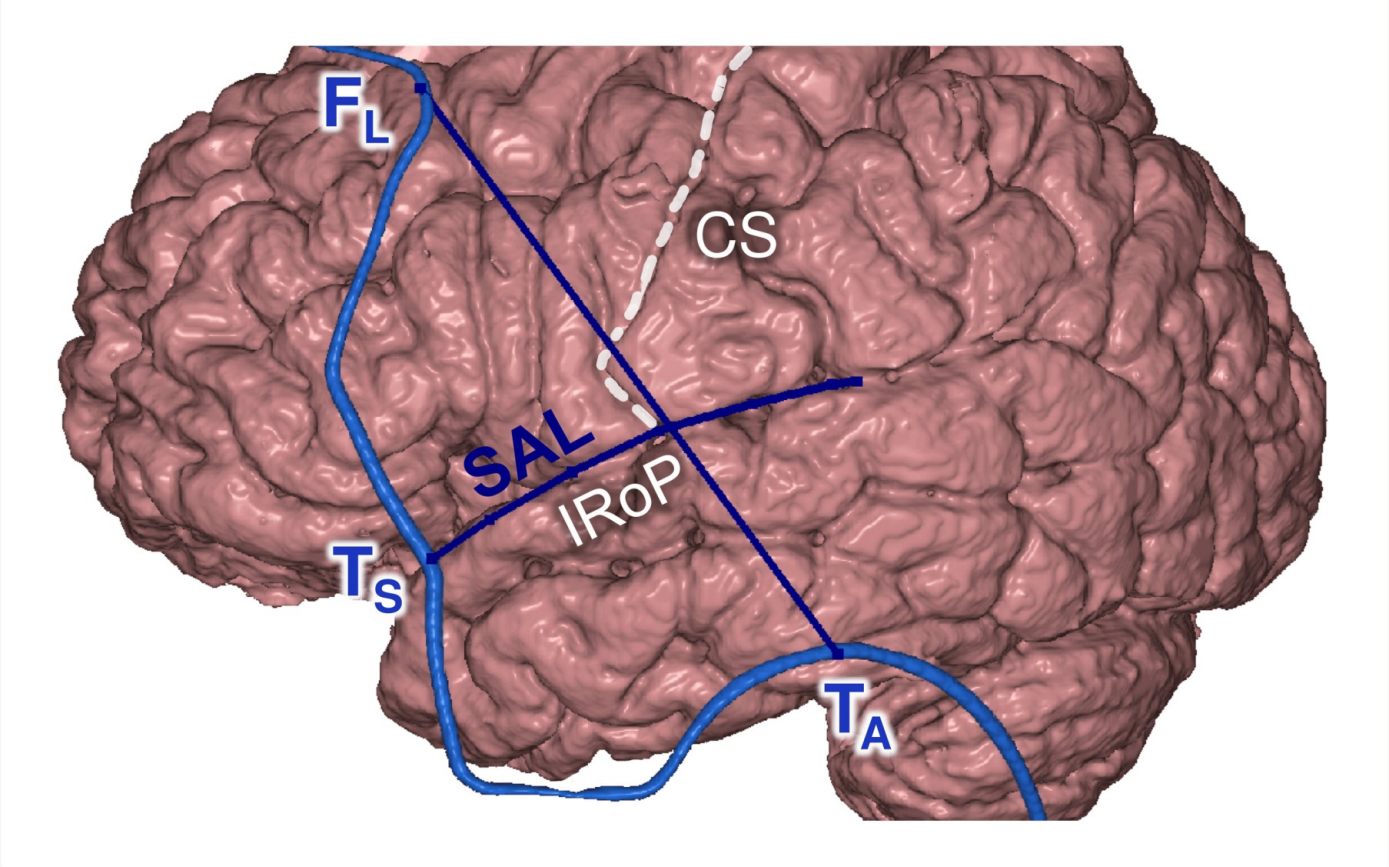
Hairline and the IRoP F_L_: lateral frontal point; T_S_: sphenoidal point; T_A_: auricular point; SAL: supraauricular line; CS: central sulcus; IRoP: inferior rolandic point This figure has been created by the authors using Inobitec PRO software (version 2.15.2; Inobitec Software, Voronezh, Russia) and Microsoft PowerPoint (Microsoft Corporation, Redmond, WA, United States).

Intersection of SAL and F_L_-T_A_ line was projected on the inferior rolandic point (IRoP) (shift 5.6±4.1 mm, category B) (Figure 14). IRoP is the SF intersection with the central sulcus, which serves for determining the precentral and postcentral gyri, as well as the Heschl gyrus, the lateral end of which is located immediately posterior to IRoP, under the postcentral gyrus [5-7,37].

Occipital hairline topography

The O_M _Point

**Figure 15:**
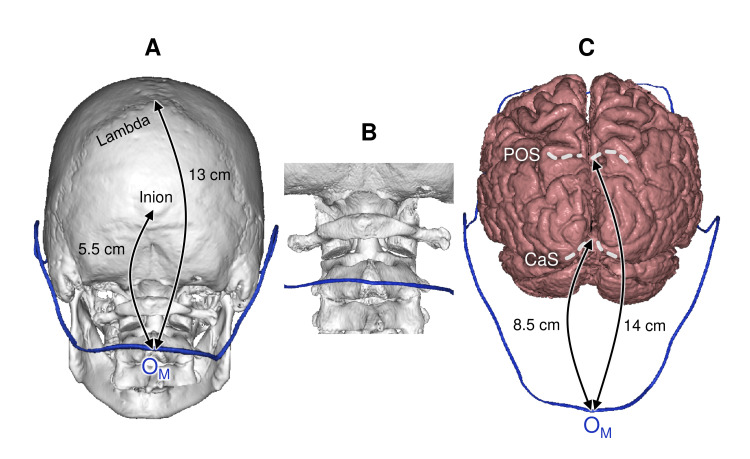
Median occipital point topography (A) Projection on the skull bones; (B) Projection on the C2 spinal process; (C) Projection on the cerebral cortex O_M_: median occipital point; POS: parieto-occipital sulcus; CaS: calcarine sulcus This figure has been created by the authors using Inobitec PRO software (version 2.15.2; Inobitec Software, Voronezh, Russia) and Microsoft PowerPoint (Microsoft Corporation, Redmond, WA, United States).

The O_M_ point was most often located at the level of the C2 spinal process (shift 10.5±7.5 mm, category C). The O_M_-inion distance was 5.5 cm (shift 10.7±7.4 mm, category C); O_M_-lambda distance - 13 cm (shift 10.5±8.3 mm, category C). The distance from the O_M_ point to the calcarine sulcus was 8.5 cm (shift 14.8±8.3 mm, category D); to the parieto-occipital sulcus, 14 cm (shift 13.6±9.0 mm, category D) (Figure 15).

Hairline landmarks accuracy

Quantitatively, the overall landmark-target shift was 6.9±3.3 (0.3-14.8) mm. The shift significantly depended on the hairline region (p<0.001) and increased respectively as follows: temporal region, 5.0±2.3 (0.3-10.1) mm; frontal, 7.3±1.9 (3.7-9.2) mm; occipital, 12.0±2.0 (10.5-14.8) mm.

Qualitatively, the accuracy was as follows: category A, two (7.7%) landmarks; B, 13 (51.9%) landmarks; C, 9 (32.7%) landmarks; D, two (7.7%) landmarks. Distribution by the hairline regions was as follows: Frontal region: category B, two (28.6%) landmarks; C, five (71.4%) landmarks. Temporal region: A, two (13.3%) landmarks; B, 12 (80%) landmarks; C, one (6.7%) landmark. Occipital region: C, three (60%) landmarks; D, two (40%) landmarks.

## Discussion

What do we know about the hair?

In neurosurgical practice, there were two concepts associated with hair. The first is the classic dogma “not to cut the skin outside the hairline”, in order to avoid cosmetic deficiency [38]. The second is the discussion about the need for preoperative hair shaving [39-41]. In the rest, the hair remained just an irritating obstacle, which conceals the landmarks and hinders the wound suturing.

In this study, we tried to change this conception and find out if it is possible to benefit from hair by using it as a landmark.

Use of the hairline

In this work, we have discovered the relation of the hairline to more than 20 important neuroanatomical structures. This information can be useful in several ways.

Firstly, knowledge of the identified landmarks improves overall anatomical orientation by better understanding of the important structures' projections on the head surface. This simplifies the preoperative planning of neurosurgical approaches.

Secondly, the hairline can serve as a guide for designing skin incisions. The formulated names of the hairline parts allow to briefly describe the course of incision, which is especially useful at the initial stage of approach learning. It is much easier to remember and repeat the incision line when its beginning and end are directly visible and naturally marked by the hairline.

Thirdly, this information can be used in neurophysiology, namely in transcranial magnetic stimulation (TMS) [42], transcranial direct current stimulation [43], and transcranial motor evoked potentials [44], when it is necessary to localize the target cortical area for the correct placement of the coil or electrodes.

The main advantage of the hairline as a landmark is its absolute accessibility: it is clearly visible from afar, even after hair shaving.

The following are specific examples of possible hairline uses obtained by comparing new data with traditional literature descriptions.

Design of the skin incisions

**Figure 16:**
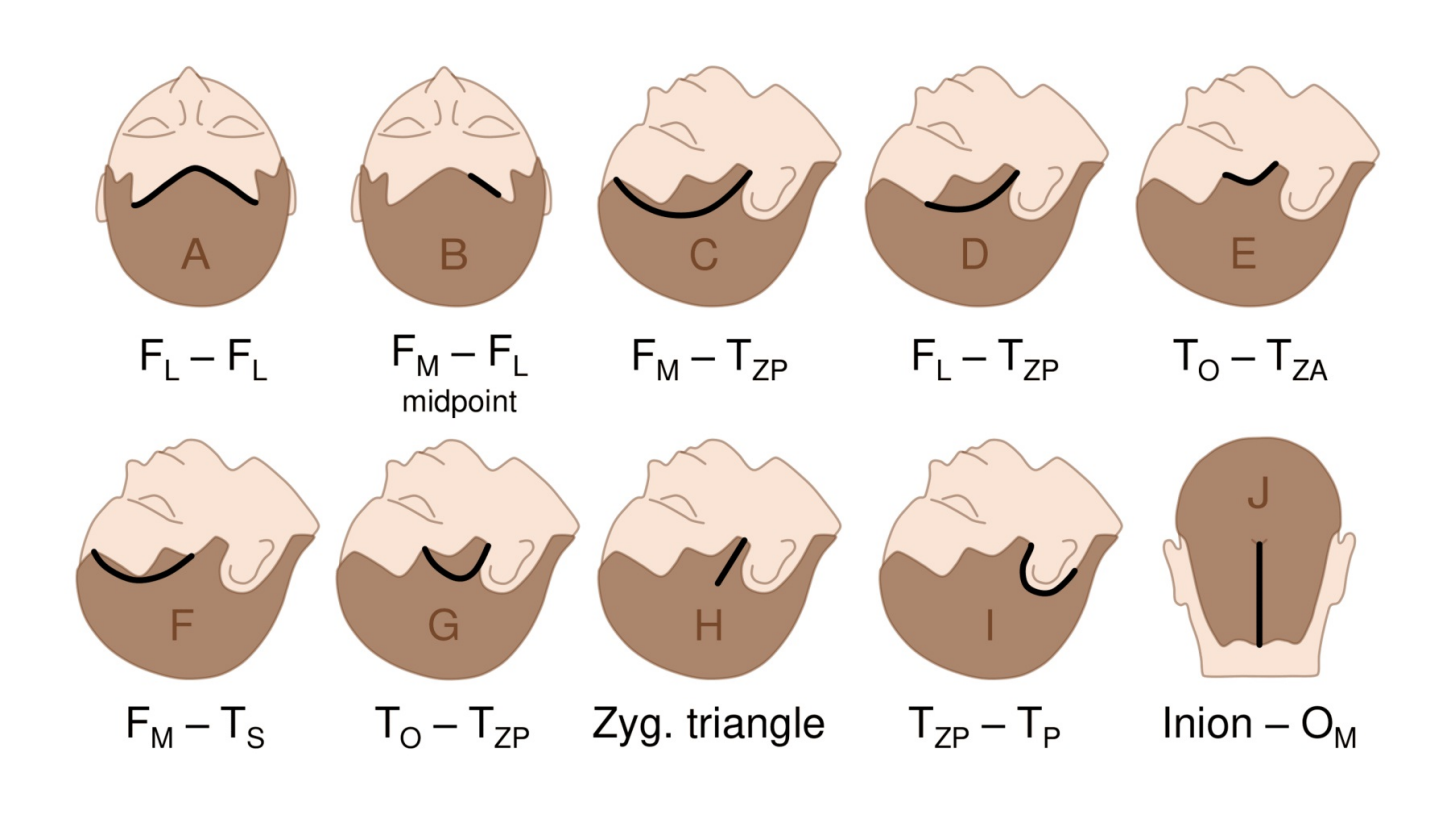
Skin incisions using the hairline, in terms of described hair points (A) Anterior interhemispheric approach [45]; (B) Endoscopic approach to the third ventricle and sylvian aqueduct [46]; (C) Pterional approach [47]; (D) Pterional approach within the temporal fossa [48,49]; (E) Mini-pterional approach [50,51]; (F) Lateral supraorbital approach [52–54]; (G) Mini-temporal approach [55]; (H) Anterior subtemporal keyhole approach [56,57]; (I) Subtemporal approach [53,58]; (J) Median suboccipital approach [45,53] FL: lateral frontal point; F_M_: median frontal point; T_ZP_: posterior zygomatic point; T_O_: orbital point; T_ZA_: anterior zygomatic point; T_S_: sphenoidal point; T_P_: petrosal point; O_M_: median occipital point This figure has been created by the authors using Microsoft PowerPoint (Microsoft Corporation, Redmond, WA, United States).

A number of classical and minimalistic neurosurgical approaches are known, in which skin incisions run along or parallel to the hairline [45-58]. They can be rethought and reformulated, given the new data; the information is schematically presented in Figure 16.

Search for vessels for revascularization

The STA frontal branch and stem can be detected by palpation or Doppler scanning along the T_O_-T_S_ segment and the T_ZP_ point, respectively. This may simplify their search when bypass planning [59].

The posterior end of SAL indicates the PSP location with large MCA branches, so it can be used to plan a craniotomy for STA-MCA bypass [33,34].

Facial nerve preservation

The frontal branch of the facial nerve always lies 1-2 cm anterior to the stem and frontal branch of STA [60-63]. Although we did not have the opportunity to directly observe the facial nerve (only the artery), given this information, it can be assumed that the temporal hair region is a “safe zone” with a reduced risk of nerve damage during the skin incision. The most likely anterior boundary of the safe zone is the sphenoidal angle (T_O_-T_S_-T_ZA_ segment). This hypothesis should be tested on cadaveric samples.

Eloquent cortex localization

An approximate localization of functional cortical areas is possible, given the known data on their usual position (Table 2).

**Table 2:** Hairline and the eloquent cortical areas A1: primary auditory area; DLPFC: dorsolateral prefrontal cortex; FEF: frontal eye field; IFG: inferior frontal gyrus; IRoP: inferior rolandic point; M1: primary motor area; MFG: middle frontal gyrus; S1: primary sensory area; SAL: supraauricular line; SRoP: superior rolandic point; V1: primary visual area

Functional area	Usual cortical localization	Hair landmark
DLPFC	MFG middle third [17,18]	F_L_, more precisely in females
FEF	MFG posterior third [14–16]	F_L_, more precisely in males
M1, S1	Anterior and posterior to the central sulcus [6,7]	SRoP – 12±1 cm posterior to F_M_; IRoP – F_L_–T_A_ and SAL intersection
A1	Heschl gyrus, which projects on the cortical surface just posterior to IRoP [5–7,31]	F_L_–T_A_ and SAL intersection
V1	Calcarine sulcus [6,7]	8.5±1.5 cm superior to O_M_
Broca	IFG posterior third, usually on the left [19–22]	Orbital triangle
Wernicke	Posterior perisylvian cortex, usually on the left [29,30]	SAL posterior end

Is the hairline reliable?

Is it possible to treat hair as a constant, stably located neuroanatomical structure? There are several arguments from various fields in support of this concept.

Biologically, the brain and hair (as the skin derivative) have a common origin from the ectoderm [64,65] and a pronounced biochemical interaction [66,67], which is why some authors consider them as a single physiological system.

From the genetic viewpoint, the shape, color, and texture of hair are strictly programmed, while many parameters are inherited in an autosomal dominant type [68-71].

Trichological studies indicate the existence of a common hairline pattern underlying all individual variants [10-13]. According to the largest series [10-12,17-19], the occipital and temporal hairline regions are constant in the absolute majority of people (about 95%). The frontal region depends on the sex. Intact frontal patterns occur in 98% to 99% of females and 78% to 93% of males. This is confirmed by our analysis of patients from the first group.

In trauma surgery, it was recently discovered that the axillary hairline corresponded to an area between the second and fifth intercostal spaces, so its lower edge turned out to be an acceptable landmark for thoracostomy [72].

In our work, most of the identified landmarks showed relatively high, clinically acceptable spatial accuracy, mainly categories B and C, with an overall shift of 6.9±3.3 mm. The maximum accuracy was noted in the temporal region (mean shift 5 mm) compared to the frontal (7.3 mm) and occipital (12 mm) regions. This can be possibly explained by the fact that the temporal landmarks were located bilaterally and therefore made up twice as large a sample as the frontal and occipital ones, which were located mainly on the midline. This also correlates with data on the occurrence of androgenic alopecia, which is predominant in the frontal and occipital regions.

According to the distribution of hairline patterns in the first group, as well as the indicated literature data, temporal and occipital landmarks can be used in 95% to 100% of patients of both sexes; frontal landmarks can be used in 85% of males and 99% of females.

Given the above, our usual “trust” in neuroanatomy, which generally seems stable (despite some variability), can be transferred to hair.

Limitations

The frontal hairline in 15% of males may be atrophied and unsuitable for navigation. Occipital and temporal regions are changed much less frequently, in about 5% of cases. For the correct use of landmarks, it is necessary to exclude defective patterns. This requires familiarity with the BASP classification.

Even in the absence of alopecia, individual variability may occur; in doubtful cases, it is advisable to use standard anatomical landmarks and neuronavigation.

The hairline may shift after the head fixation in the skull clamp. The described landmarks can only be used until the scalp flap retraction; after that, they are not visible.

The second part of our pilot study was conducted on a relatively modest number of patients (40 head sides), due to strict inclusion criteria, non-standard neuroimaging, and budget restrictions. However, in the field of neurosurgical craniometry, our sample can be considered adequate (see the Sample size estimation in Methods). We believe that increasing the sample will not dramatically affect the identified relationships, but may only clarify some statistics.

Data on the localization of functional areas are based on indirect information about their typical anatomical representation and are therefore speculative.

Future directions

In the future, it is desirable to expand the sample. This may be facilitated by new CT or MRI protocols for hair visualization. It is advisable to explore the children and the patients of different races (Caucasian patients predominated in our sample).

A cadaveric study of the hairline position relative to the cutaneous nerves is necessary. Further validation of the found landmarks is necessary - cadaveric, neurophysiological (using TMS), and surgical (using frameless navigation or augmented reality).

## Conclusions

This pilot study presents a novel look at the hairline from a neurosurgical viewpoint. The topographic relationship between the hairline and more than 20 neuroanatomical structures has been discovered. Specifically, the hairline allows to localize the skull base and vault structures (coronal and lambdoid sutures, sphenoidal and petrosal ridges), cervical vertebrae (C2), cortical sulci (Sylvian, central, precentral, superior, and inferior frontal), gyri (precentral, postcentral, all major frontal and temporal), and vessels (STA, sinodural angle, TSSJ).

The revealed landmarks showed acceptable spatial accuracy and representativeness. All the hairline parts (except for the frontal region in men) were fairly stable in the vast majority of patients. Therefore, the hairline can be recognized as a relatively constant structure, which is topographically connected with deep layers. The new data can be used in medical practice for tasks related to the projection of neuroanatomic structures onto the scalp, such as planning neurosurgical approaches, placing neurophysiological devices over functional cortical areas, and so on.

## Figures and Tables

**Figure 17:**
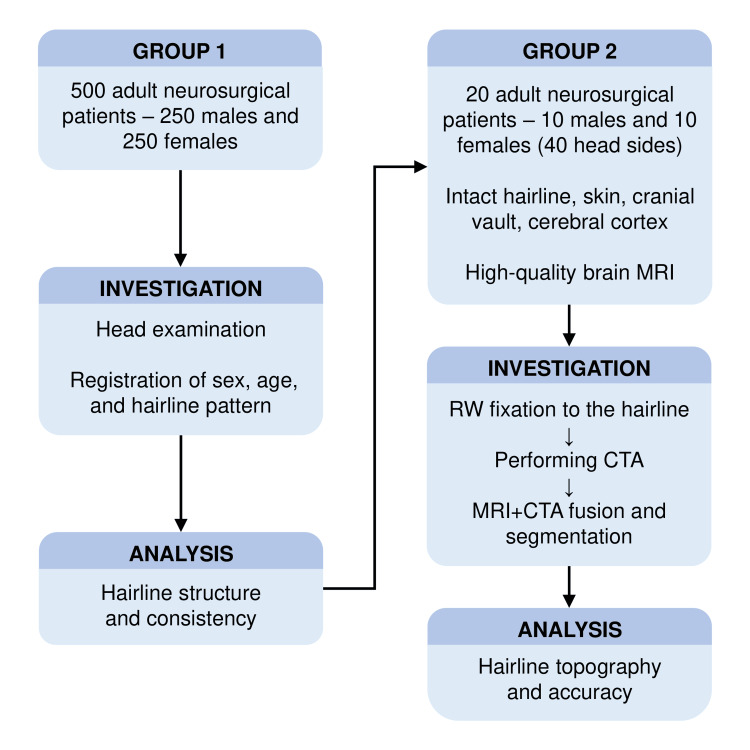
Study design MRI: magnetic resonance imaging; CTA: computed tomographic angiography; RW: radiopaque wire This figure has been created by the authors using Microsoft PowerPoint (Microsoft Corporation, Redmond, WA, United States).

**Figure 18:**
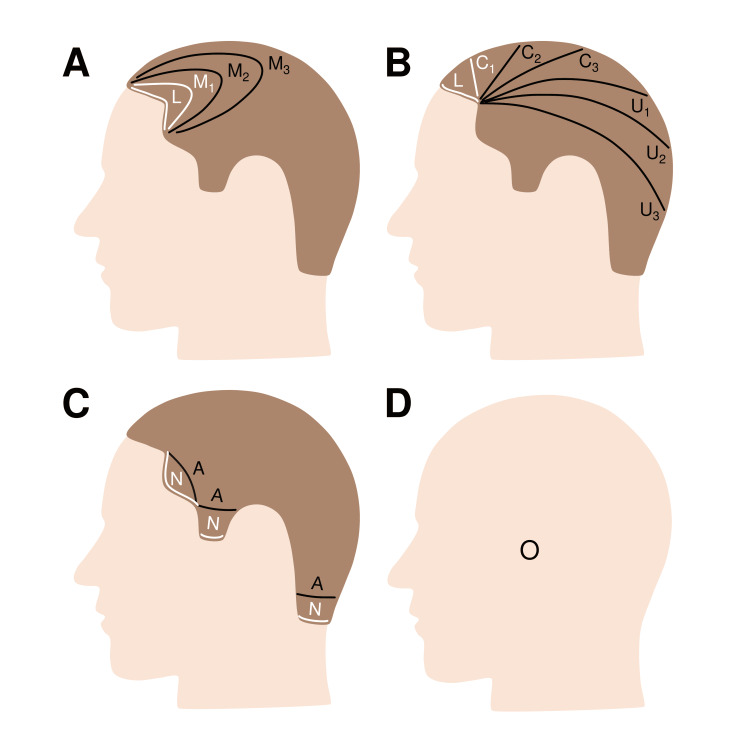
Modified Basic and Specific (BASP) classification (A) M-shaped frontal patterns of the hairline, White: norm (L, M1), Black: atrophy (M2, M3); (B) C and U-shaped frontal patterns, White: norm (L, C1), Black: atrophy (C2, C3, U1, U2, U3); (C) Temporal and occipital patterns, White: norm (N); Black: atrophy (A); (D) Total alopecia (O-pattern) This figure has been created by the authors using Microsoft PowerPoint (Microsoft Corporation, Redmond, WA, United States).

**Figure 19:**
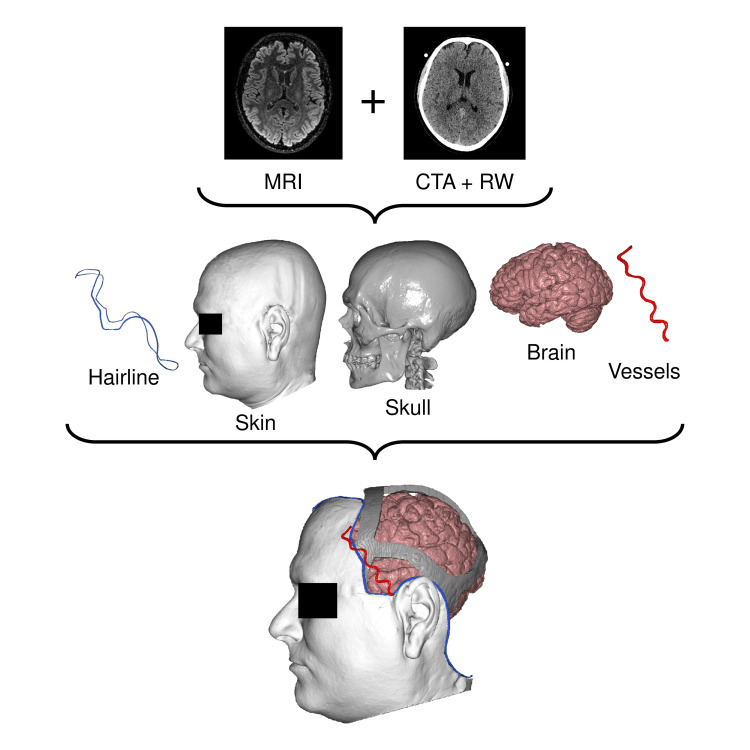
Anatomical structures visualization MRI: magnetic resonance imaging; CTA: computed tomographic angiography; RW: radiopaque wire This figure has been created by the authors using Inobitec PRO software (version 2.15.2; Inobitec Software, Voronezh, Russia) and Microsoft PowerPoint (Microsoft Corporation, Redmond, WA, United States).

**Figure 20:**
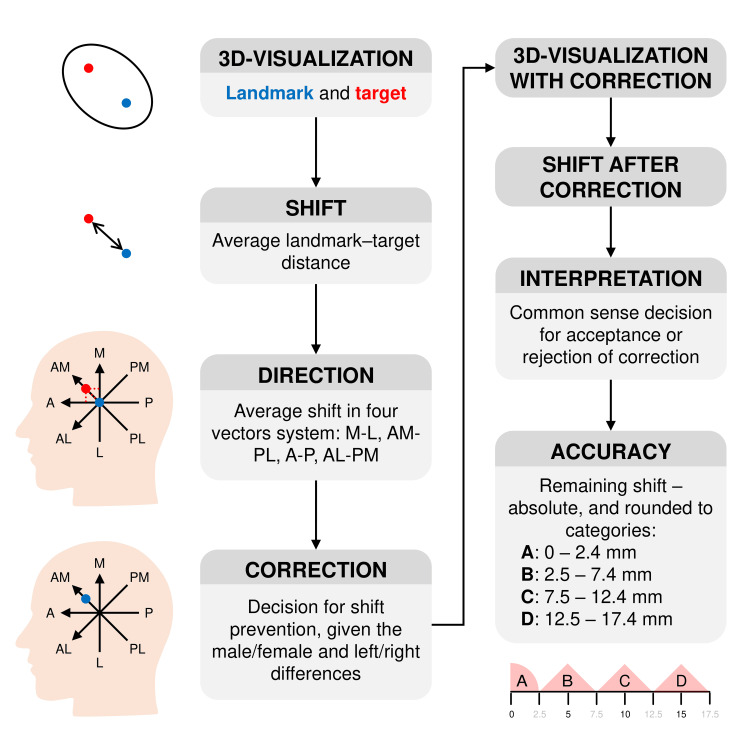
Flowchart of topographic analysis This figure has been created by the authors using Microsoft PowerPoint (Microsoft Corporation, Redmond, WA, United States).

**Figure 21:**
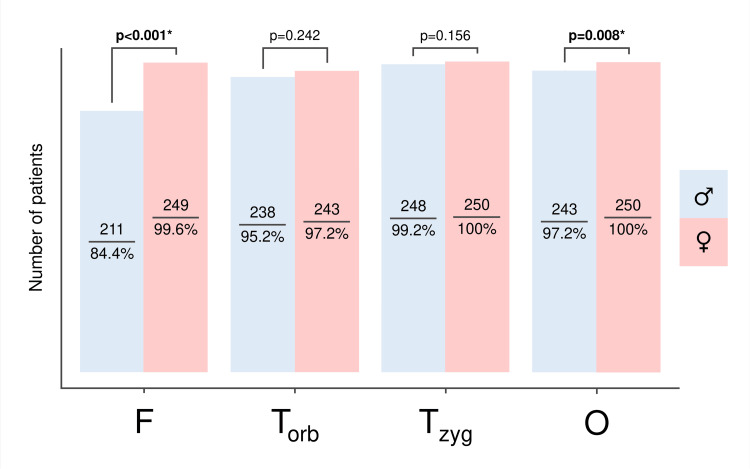
Occurrence of normal hairline patterns, depending on sex F: frontal region; T_orb_ and T_zyg_: orbital and zygomatic triangles of the temporal region; O: occipital region; *statistically significant differences This figure has been created by the authors using Microsoft PowerPoint (Microsoft Corporation, Redmond, WA, United States).

## Appendices

Appendix A 

Appendix B 

Appendix C

Appendix D 

Appendix E 

Appendix F 

**Table 3:** Qualitative topographic analysis of the hairline Projections on the neuroanatomical structures (number and % of cases); Male/female and left/right differences: *statistically significant (p<0.05), **statistically and clinically significant; CorSut: coronal suture; F_L_: lateral frontal point; F_M_: median frontal point; IFG: inferior frontal gyrus; IFS: inferior frontal sulcus; ITG: inferior temporal gyrus; ITS: inferior temporal sulcus; MFG: middle frontal gyrus; MTG: middle temporal gyrus. O_M_: median occipital point; SFG: superior frontal gyrus; SF_H_: Sylvian fissure, horizontal branch; SFS: superior frontal sulcus; STG: superior temporal gyrus; STS: superior temporal sulcus; T_A_: auricular point; T_O_: orbital point; T_S_: sphenoidal point; T_ZA_: anterior zygomatic point; T_ZP_: posterior zygomatic point

Topography	Structure	Total	Male	Female	Left	Right
1. F_M_ – bregma	F_M_ is anterior to bregma	20 (100%)	20 (100%)	20 (100%)	-	-
	F_M_ is at bregma	0 (0%)	0 (0%)	0 (0%)	-	-
	F_M_ is posterior to bregma	0 (0%)	0 (0%)	0 (0%)	-	-
			M=F (p=1.0)		-	
2. F_M_–F_L_ midpoint – cortex	SFG	14 (35%)	5 (25%)	9 (45%)	4 (20%)	10 (50%)
	SFS	20 (50%)	10 (50%)	10 (50%)	13 (65%)	7 (35%)
	MFG	6 (15%)	5 (25%)	1 (5%)	3 (15%)	3 (15%)
			M=F (p=0.171)		L=R (p=0.112)	
3. F_L_ – coronal suture	F_L_ is anterior to CorSut	28 (70%)	8 (40%)	20 (100%)	13 (65%)	15 (75%)
	F_L_ is at CorSut	3 (7.5%)	3 (15%)	0 (0%)	3 (15%)	0 (0%)
	F_L_ is posterior to CorSut	9 (22.5%)	9 (45%)	0 (0%)	4 (20%)	5 (25%)
			M≠F (p<0.001)**		L=R (p=0.327)	
4. F_L_ – cortex	MFG (anterior third)	3 (7.5%)	0 (0%)	3 (15%)	1 (5%)	2 (10%)
	MFG (middle third)	16 (40%)	2 (10%)	14 (70%)	9 (45%)	7 (35%)
	MFG (posterior third)	21 (52.5%)	18 (90%)	3 (15%)	10 (50%)	11 (55%)
			M≠F (p<0.001)**		L=R (p=0.802)	
6. F_L_–T_O_ midpoint – cortex	MFG	12 (30%)	6 (30%)	6 (30%)	2 (10%)	10 (50%)
	IFS	26 (65%)	13 (65%)	13 (65%)	17 (85%)	9 (45%)
	IFG	2 (5%)	1 (5%)	1 (5%)	1 (5%)	1 (5%)
			M=F (p=1.0)		L≠R (p=0.014)*	
7. T_O_ – cortex	IFG (orbital part)	9 (22.5%)	1 (5%)	8 (40%)	4 (20%)	5 (25%)
	IFG (SF_H_)	22 (55%)	12 (30%)	10 (50%)	11 (55%)	11 (55%)
	IFG (triangular part)	9 (22.5%)	7 (35%)	2 (10%)	5 (25%)	5 (25%)
			M≠F (p=0.018)**		L=R (p=1.0)	
8. T_S_ – cortex	IFG	2 (5%)	1 (5%)	1 (5%)	1 (5%)	1 (5%)
	SF stem	16 (40%)	7 (35%)	9 (45%)	6 (30%)	10 (50%)
	STG	22 (55%)	12 (60%)	10 (50%)	13 (50%)	9 (45%)
			M=F (p=0.868)		L=R (p=0.416)	
9. T_ZA_ – cortex	Temporal pole	34 (85%)	14 (70%)	20 (100%)	17 (85%)	17 (85%)
	MTG	6 (15%)	6 (30%)	0 (0%)	3 (15%)	3 (15%)
			M≠F (p=0.02)*		L=R (p=1.0)	
10. T_ZP_ – cortex	MTG	11 (27.5%)	10 (50%)	1 (5%)	6 (30%)	5 (25%)
	ITS	5 (12.5%)	4 (20%)	1 (5%)	3 (15%)	2 (10%)
	ITG	24 (60%)	6 (30%)	18 (90%)	11 (55%)	13 (65%)
			M≠F (p<0.001)*		L=R (p=0.819)	
11. T_A_ – cortex	STG (middle third)	2 (5%)	2 (10%)	0 (0%)	1 (5%)	1 (5%)
	MTG (middle third)	28 (70%)	12 (60%)	16 (80%)	15 (75%)	13 (65%)
	ITS (middle third)	8 (20%)	4 (20%)	4 (20%)	2 (10%)	6 (30%)
	ITG (middle third)	2 (5%)	2 (10%)	0 (0%)	2 (10%)	0 (0%)
			M=F (p=0.325)		L=R (p=0.294)	
12. O_M_ – vertebra	C1	1 (5%)	1 (10%)	0 (0%)	-	-
	C2	11 (55%)	7 (70%)	4 (40%)	-	-
	C3	7 (35%)	2 (20%)	5 (50%)	-	-
	C4	1 (5%)	0 (0%)	1 (10%)	-	-
			M≠F (p=0.029)*		-	

Appendix G 

**Table 4:** Quantitative topographic analysis of the hairline. Shifts, corrections, and categories Male/female and left/right differences: * statistically significant (p<0.05), **statistically and clinically significant; ASP: anterior sylvian point; CorSut: coronal suture; F_L_: lateral frontal point; F_M_: median frontal point; IFG: inferior frontal gyrus; IFS: inferior frontal sulcus; IRoP: inferior rolandic point; O_M_: median occipital point; PreCS: precentral sulcus; PRP: petrosal ridge point; PSP: posterior sylvian point; SAL: supraauricular line; SF: Sylvian fissure; SFS: superior frontal sulcus; SRP: sphenoidal ridge point; SRoP: superior rolandic point; STA: superficial temporal artery; T_A_: auricular point; T_O_: orbital point; T_S_: sphenoidal point; T_ZA_: anterior zygomatic point; T_ZP_: posterior zygomatic point

Landmark - target	Initial hypothesis	Mean shift, mm							M-L shift, mm							AM-PL shift, mm							A-P shift, mm							AL-PM shift, mm							Correction	Shift after correction, mm					Decision
.	.	Total	Male	Female	M vs F	Left	Right	L vs R	Total	Male	Female	M vs F	Left	Right	L vs R	Total	Male	Female	M vs F	Left	Right	L vs R	Total	Male	Female	M vs F	Left	Right	L vs R	Total	Male	Female	M vs F	Left	Right	L vs R	.	Total	Male	Female	Left	Right	.
1. F_M_ – nasion	F_M_ is at nasion	65.3±10.9 (51 – 91)	67.6±12.6 (52 – 91)	63.0±8.6 (51 – 75)	M=F (p=0.261)	.	.	.	.	.	.	.	.	.	.	.	.	.	.	.	.	.	65.3±10.9 (51 – 91)	67.6±12.6 (52 – 91)	63.0±8.6 (51 – 75)	M=F (p=0.261)	.	.	.	.	.	.	.	.	.	.	Nasion is 6.5 cm anterior to F_M_	8.9±6.1 (1 – 26)	10.0±7.7 (1 – 26)	7.8±3.7 (2 – 14)	.	.	Accept correction. Category С
2. F_M_ – bregma	Bregma is at F_M_	64.7±10.0 (46 – 84)	61.8±10.9 (46 – 84)	63.0±8.6 (51 – 75)	M=F (p=0.072)	.	.	.	.	.	.	.	.	.	.	.	.	.	.	.	.	.	-64.7±10.0 (-84 – -46)	-61.8±10.9 (-84 – -46)	-63.0±8.6 (-75 – -51)	M=F (p=0.072)	.	.	.	.	.	.	.	.	.	.	Bregma is 6.5 cm posterior to F_M_	8.1±5.9 (0 – 19)	8.8±6.9 (0 – 19)	7.3±4.7 (0 – 15)	.	.	Accept correction. Category С
3. F_M_ – superior rolandic point	F_M_ is at SRoP	116.8±8.7 (102 – 129)	116.1±9.0 (104 – 129)	117.6±8.5 (102 – 128)	M=F (p=0.559)	.	.	.	.	.	.	.	.	.	.	.	.	.	.	.	.	.	-116.8±8.7 (-129 – -102)	-116.1±9.0 (-129 – -104)	-117.6±8.5 (-128 – 102)	M=F (p=0.559)	.	.	.	.	.	.	.	.	.	.	SRoP is 12 cm posterior to F_M_	7.7±5.1 (0 – 18)	8.5±4.6 (0 – 16)	6.8±5.4 (0 – 18)	.	.	Accept correction. Category C
4. F_M_–F_L_ midpoint – superior frontal sulcus	SFS is at F_M_–F_L_ midpoint	3.7±3.4 (0 – 10)	3.7±3.0 (0 – 9)	3.7±3.8 (0 – 10)	M=F (p=0.944)	3.0±3.0 (0 – 9)	4.4±3.7 (0 – 10)	L=R (p=0.19)	-2.6±4.4 (-10 – 7)	-1.9±4.6 (-9 – 7)	-3.2±4.3 (-10 – 5)	M=F (p=0.427)	-1.3±4.1 (-9 – 6)	-3.8±4.5 (-10 – 7)	L=R (p=0.073)	.	.	.	.	.	.	.	.	.	.	.	.	.	.	.	.	.	.	.	.	.	SFS is 5 mm inferior to F_M_–F_L_ midpoint	3.6±2.9 (0 – 10)	3.5±3.3 (0 – 10)	3.7±2.5 (0 – 9)	.	.	No need for correction. Category B
5. F_L_ – coronal suture	CorSut is at the F_L_	15.0±10.9 (0 – 43)	8.1±5.7 (0 – 18)	21.9±10.6 (6 – 43)	M≠F (p<0.001)**	13.3±10.0 (0 – 33)	16.7±11.8 (4 – 43)	L=R (p=0.339)	.	.	.	.	.	.	.	.	.	.	.	.	.	.	-10.9±15.1 (-43 – 15)	0.2±10.0 (-18 – 15)	-21.9±10.6 (-43 – -6)	M≠F (p<0.001)**	-9.2±14.0 (-33 – 13)	-12.6±16.3 (-43 – 15)	L=R (p=0.49)	.	.	.	.	.	.	.	Male: CorSut is at F_L_. Female: CorSut is 2 cm posterior to F_L_.	8.6±5.7 (0 – 24)	8.1±5.7 (0 – 18)	9.2±5.7 (0 – 24)	.	.	Accept correction. Category C
6. F_L_ – precentral sulcus	PreCS is at F_L_	22.0±14.0 (3 – 45)	10.6±7.4 (3 – 30)	33.4±8.6 (15 – 45)	M≠F (p<0.001)**	23.0±14.3 (3 – 45)	20.9±14.0 (3 – 43)	L=R (p=0.649)	.	.	.	.	.	.	.	.	.	.	.	.	.	.	-22.0±14.0 (-45 – -3)	-10.6±7.4 (-30 – 3)	-33.4±8.6 (-45 – -15)	M≠F (p<0.001)**	-23.0±14.3 (-45 – -3)	-20.9±14.0 (-43 – -3)	L=R (p=0.649)								Male: PreCS is 1 cm posterior to F_L_. Female: PreCS is 3.5 cm posterior to F_L_.	6.5±6.9 (0 – 25)	5.0±6.3 (0 – 22)	8.0±7.3 (0 – 25)	.	.	Accept correction. Male – category B, female – category C
7. F_L_–T_O_ midpoint – inferior frontal sulcus	IFS is at F_L_–T_O _midpoint	2.2±2.7 (0 – 10)	2.0±2.3 (0 – 6)	2.4±3.1 (0 – 10)	M=F (p=0.721)	1.1±2.0 (0 – 6)	3.3±2.9 (0 – 10)	L≠R (p=0.008)*	-1.4±3.2 (-10 – 6)	-1.4±2.7 (-5 – 6)	-1.5±3.6 (-10 – 6)	M=F (p=0.976)	-0.2±2.2 (-5 – 6)	-2.7±3.5 (-10 – 6)	L≠R (p=0.005)*	.	.	.	.	.	.	.	.	.	.	.	.	.	.	.	.	.	.	.	.	.	On the right, IFS is 5 mm inferior to F_L_–T_O_ midpoint	1.6±2.0 (0 – 6)	.	.	1.1±2.0 (0 – 6)	2.2±1.9 (0 – 5)	No need for correction. Category A
8. T_O_ – inferior frontal gyrus, triangular part center	IFG triangular part center is at T_O_	10.9±7.4 (0 – 30)	6.9±4.8 (0 – 17)	14.9±7.4 (0 – 30)	M≠F (p<0.001)**	10.4±7.8 (0 – 30)	11.4±7.1 (0 – 22)	L=R (p=0.674)	1.1±6.6 (-15 – 15)	0.2±4.0 (-7 – 11)	2.1±8.5 (-16 – 16)	M=F (p=0.371)	2.7±6.2 (-11 – 16)	-0.5±6.9 (-16 – 15)	L=R (p=0.128)	-4.7±7.3 (-22 – 12)	-2.0±5.7 (-10 – 12)	-7.4±7.9 (-22 – 0)	M≠F (p=0.016)*	-3.9±6.6 (-21 – 8)	-5.6±8.1 (-22 – 12)	L=R (p=0.465)	-7.8±8.4 (-30 – 17)	-3.0±6.9 (-13 – 17)	-12.5±6.9 (-30 – 0)	M≠F (p<0.001)**	-8.1±7.7 (-30 – 6)	-7.4±9.2 (-22 – 17)	L=R (p=0.781)	-6.3±7.8 (-22 – 12)	-2.2±5.6 (-15 – 12)	-10.3±7.6 (-22 – 0)	M≠F (p<0.001)*	-7.7±7.3 (-22 – 0)	-4.9±8.1 (-21 – 12)	L=R (p=0.262)	Male: IFG triangular part center is 5 mm posterior to T_O_. Female: IFG triangular part center is 15 mm posterior to T_O_	5.9±3.3 (0 – 11)	4.9±3.4 (0 – 10)	7.0±3.0 (0 – 11)	.	.	Accept correction. Category B
9. T_O_–T_S_ – superficial temporal artery, frontal branch	STA frontal branch is at T_O_–T_S_	6.7±4.9 (0 – 18)	7.4±5.0 (0 – 18)	5.9±4.9 (0 – 17)	M=F (p=0.323)	6.5±5.4 (0 – 18)	6.8±4.5 (0 – 15)	L=R (p=0.842)	.	.	.	.	.	.	.	.	.	.	.	.	.	.	.	.	.	.	.	.	.	4.6±6.9 (-8 – 18)	5.9±6.8 (-6 – 18)	3.2±7.0 (-8 – 17)	M=F (p=0.226)	4.7±7.1 (-6 – 18)	4.4±7.0 (-8 – 15)	L=R (p=0.895)	STA frontal branch is 5 mm anteroinferior to T_O_–T_S_	5.2±3.7 (0 – 13)	4.8±3.8 (0 – 12)	5.6±3.6 (1 – 13)	.	.	No need for correction (clinically insignificant). Category B
10. T_S_ – sphenoidal depression	Sphenoidal depression is at T_S_	6.8±4.7 (0 – 15)	6.9±4.4 (0 – 13)	6.7±5.1 (0 – 15)	M=F (p=0.895)	7.8±4.2 (0 – 14)	5.7±5.0 (0 – 15)	L=R (p=0.161)	.	.	.	.	.	.	.	6.1±5.5 (-7 – 15)	6.2±5.4 (-7 – 13)	6.1±5.8 (-6 – 15)	M=F (p=0.955)	6.5±6.1 (-7 – 14)	5.7±5.0 (0 – 15)	L=R (p=0.654)	.	.	.	.	.	.	.	.	.	.	.	.	.	.	Sphenoidal depression is 5 mm anterosuperior to T_S_	3.2±3.0 (0 – 13)	3.3±3.5 (0 – 13)	3.2±2.6 (0 – 8)	.	.	No need for correction (clinically insignificant). Category B
11. T_S_ – sphenoidal ridge point	SRP is at T_S_	10.1±4.9 (0 – 20)	11.3±4.7 (5 – 20)	9.0±5.0 (0 – 20)	M=F (p=0.143)	10.0±5.0 (0 – 20)	10.3±5.0 (0 – 20)	L=R (p=0.851)	2.8±7.0 (-14 – 18)	0.9±7.3 (-14 – 11)	5.5±5.6 (-7 – 18)	M≠F (p=0.011)*	2.6±6.5 (-14 – 18)	3.0±7.5 (-14 – 14)	L=R (p=0.876)	4.6±5.6 (-10 – 15)	6.4±4.8 (0 – 15)	2.8±5.9 (-10 – 13)	M≠F (p=0.044)*	5.8±5.2 (-6 – 13)	3.4±5.9 (-10 – 15)	L=R (p=0.187)	3.7±7.7 (-14 – 18)	8.9±4.5 (0 – 18)	-1.6±6.7 (-14 – 9)	M≠F (p<0.001)*	5.5±6.9 (-9 – 18)	1.8±8.2 (-14 – 14)	L=R (p=0.133)	0.6±8.7 (-20 – 20)	6.2±7.1 (-5 – 20)	-5.0±6.4 (-20 – 5)	M≠F (p<0.001)*	2.0±8.0 (-13 – 20)	-0.8±9.4 (-20 – 20)	L=R (p=0.306)	Male: SRP is 10 mm anterior to T_S_. Female: SRP is 5 mm superior to T_S_	7.2±4.1 (0 – 15)	5.4±4.1 (0 – 15)	8.9±3.3 (2 – 15)	.	.	No need for correction (clinically insignificant). Category C
12. T_S_ – sylvian fissure stem	SF stem is at T_S_	0.3±2.8 (0 – 8)	2.5±2.9 (0 – 7)	2.1±2.7 (0 – 8)	M=F (p=0.656)	3.2±3.2 (0 – 8)	1.4±2.1 (0 – 6)	L=R (p=0.066)	.	.	.	.	.	.	.	1.6±2.8 (-4 – 8)	1.7±2.7 (0 – 7)	1.6±3.0 (-4 – 8)	M=F (p=0.870)	2.6±3.1 (0 – 8)	0.7±2.3 (-4 – 6)	L=R (p=0.051)	.	.	.	.	.	.	.	.	.	.	.	.	.	.	.	.	.	.	.	.	No need for correction. Category A
13. T_S_ – anterior sylvian point	ASP is at T_S_	9.3±5.1 (0 – 20)	7.8±4.2 (0 – 16)	10.9±5.5 (0 – 20)	M=F (p=0.05)	9.6±4.9 (0 – 20)	9.1±5.4 (0 – 17)	L=R (p=0.784)	6.0±6.8 (-10 – 20)	4.5±5.9 (-9 – 16)	7.4±7.4 (-10 – 20)	M=F (p=0.17)	7.1±5.8 (-5 – 20)	4.8±7.7 (-10 – 16)	L=R (p=0.303)	4.3±5.9 (-14 – 14)	5.0±4.8 (-6 – 12)	3.6±6.9 (-14 – 14)	M=F (p=0.434)	6.2±5.0 (0 – 14)	2.4±6.3 (-14 – 11)	L≠R (p=0.04)*	0.1±5.8 (-12 – 10)	2.7±4.2 (-7 – 10)	-2.4±6.0 (-12 – 9)	M≠F (p=0.007)*	1.7±5.6 (-10 – 9)	-1.5±5.6 (-12 – 10)	L=R (p=0.084)	-4.1±6.7 (-17 – 7)	-1.3±5.4 (-11 – 7)	-7.0±6.7 (-17 – 5)	M≠F (p=0.006)*	-3.8±6.4 (-14 – 7)	-4.4±7.1 (-17 – 7)	L=R (p=0.769)	ASP is 5 mm superior to T_S_	5.2±3.8 (0 – 15)	6.3±4.4 (0 – 15)	4.1±2.7 (0 – 10)	.	.	Accept correction. Category B
14. T_ZA_ – temporal tip	Temporal tip is at T_ZA_	8.7±5.2 (0 – 18)	9.2±3.5 (5 – 17)	8.3±6.6 (0 – 18)	M=F (p=0.613)	9.0±5.2 (0 – 18)	8.5±5.4 (0 – 18)	L=R (p=0.789)	.	.	.	.	.	.	.	.	.	.	.	.	.	.	8.7±5.2 (0 – 18)	9.2±3.5 (5 – 17)	8.3±6.6 (0 – 18)	M=F (p=0.613)	9.0±5.2 (0 – 18)	8.5±5.4 (0 – 18)	L=R (p=0.789)	.	.	.	.	.	.	.	Temporal tip is 1 cm anterior to T_ZA_	4.1±3.4 (0 – 10)	2.8±2.2 (0 – 7)	5.5±3.9 (0 – 10)	4.2±3.2 (0 – 10)	4.1±3.7 (0 – 10)	Accept correction. Category B
15. T_ZP _– temporal tip	Temporal tip is at T_ZP_	26.8±4.6 (16 – 39)	28.3±2.8 (21 – 33)	25.4±5.6 (16 – 39)	M≠F (p=0.009)*	26.3±4.3 (17 – 34)	27.4±5.0 (16 – 39)	L=R (p=0.44)	.	.	.	.	.	.	.	.	.	.	.	.	.	.	26.8±4.6 (16 – 39)	28.3±2.8 (21 – 33)	25.4±5.6 (16 – 39)	M≠F (p=0.009)*	26.3±4.3 (17 – 34)	27.4±5.0 (16 – 39)	L=R (p=0.44)	.	.	.	.	.	.	.	Temporal tip is 2.5 cm anterior to T_ZP_	3.9±3.1 (0 – 14)	3.8±2.0 (0 – 8)	4.0±3.9 (0 – 14)	3.6±2.7 (0 – 9)	4.2±3.5 (0 – 14)	Accept correction. Category B
16. T_ZP_ – superficial temporal artery, stem	STA stem is at T_ZP_	3.2±3.0 (0 – 9)	3.6±3.2 (0 – 9)	2.9±2.9 (0 – 7)	M=F (p=0.583)	2.8±3.0 (0 – 9)	3.6±3.1 (0 – 9)	L=R (p=0.398)	.	.	.	.	.	.	.	.	.	.	.	.	.	.	-3.1±3.1 (-9 – 0)	-3.6±3.2 (-9 – 0)	-2.6±2.9 (-7 – 0)	M=F (p=0.404)	-2.8±3.0 (-9 – 0)	-3.4±3.2 (-9 – 0)	L=R (p=0.581)	.	.	.	.	.	.	.	STA stem is 5 mm posterior to T_ZP_	1.7±2.4 (0 – 10)	2.6±2.7 (0 – 10)	0.9±1.6 (0 – 5)	.	.	No need for correction (clinically insignificant). Category B
17. T_P_ – petrous ridge point	PRP is at T_P_	6.4±4.5 (0 – 15)	6.9±4.7 (0 – 15)	5.9±4.4 (0 – 13)	M=F (p=0.488)	6.2±4.6 (0 – 15)	6.5±4.5 (0 – 15)	L=R (p=0.835)	2.0±4.4 (-7 – 13)	2.5±4.7 (-6 – 13)	1.5±4.1 (-7 – 12)	M=F (p=0.494)	2.4±4.8 (-7 – 13)	1.6±3.9 (-6 – 11)	L=R (p=0.559)	1.0±5.6 (-11 – 12)	2.0±5.6 (-11 – 10)	0.0±5.5 (-9 – 12)	M=F (p=0.273)	1.5±5.7 (-11 – 9)	0.6±5.5 (-9 – 12)	L=R (p=0.630)	-0.5±6.2 (-15 – 9)	0.4±6.6 (-15 – 9)	-1.5±5.8 (-13 – 9)	M=F (p=0.364)	-0.4±5.7 (-15 – 7)	-0.7±6.8 (-13 – 9)	L=R (p=0.842)	-1.8±5.1 (-15 – 6)	-1.5±5.8 (-15 – 6)	-2.1±4.5 (-10 – 5)	M=F (p=0.721)	-2.0±4.8 (-11 – 5)	-1.6±5.5 (-15 – 6)	L=R (p=0.851)	.	.	.	.	.	.	No need for correction. Category B
18. T_P_ – transverse-sigmoid sinus junction	TSSJ is at T_P_	12.1±3.5 (7 – 20)	11.9±3.2 (7 – 19)	12.4±3.8 (8 – 20)	M=F (p=0.924)	12.1±3.6 (7 – 19)	12.2±3.5 (7 – 20)	L=R (p=0.734)	0.3±0.9 (0 – 4)	0.3±0.8 (0 – 3)	0.4±1.1 (0 – 4)	M=F (p=0.959)	0.5±1.2 (0 – 4)	0.2±0.7 (0 – 3)	L=R (p=0.311)	-8.6±2.5 (-14 – -5)	-8.5±2.3 (-14 – -5)	-8.8±2.7 (-14 – -6)	M=F (p=0.924)	-8.6±2.6 (-14 – -5)	-8.7±2.4 (-14 – -5)	L=R (p=0.734)	-12.1±3.5 (-20 – -7)	-11.9±3.2 (-19 – -7)	-12.4±3.8 (-20 – -8)	M=F (p=0.924)	-12.1±3.6 (-19 – -7)	-12.2±3.4 (-20 – -7)	L=R (p=0.734)	-8.6±2.5 (-14 – -5)	-8.5±2.3 (-14 – -5)	-8.8±2.7 (-14 – -6)	M=F (p=0.695)	-8.6±2.6 (-14 – -5)	-8.7±2.4 (-14 – -5)	L=R (p=0.428)	TSSJ is 10 mm posterior to T_P_	4.1±2.8 (0 – 10)	4.3±2.9 (0 – 10)	4.0±2.8 (0 – 10)	.	.	Accept correction. Category B
19. SAL – sylvian fissure	SF is at SAL	3.6±4.7 (0 – 15)	5.7±5.4 (0 – 15)	1.5±2.7 (0 – 8)	M≠F (p=0.005)*	3.8±5.0 (0 – 15)	3.3±4.5 (0 – 13)	L=R (p=0.921)	-0.8±5.9 (-15 – 13)	-0.4±7.9 (-15 – 13)	-1.2±2.8 (-8 – 1)	M=F (p=0.854)	-1.1±6.2 (-15 – 12)	-0.5±5.7 (-9 – 13)	L=R (p=0.876)	.	.	.	.	.	.	.	.	.	.	.	.	.	.	.	.	.	.	.	.	.	.	.	.	.	.	.	No need for correction. Category B
20. SAL posterior end – posterior sylvian point	PSP is at SAL posterior end	6.4±5.4 (0 – 20)	6.5±5.3 (0 – 20)	6.3±5.6 (0 – 17)	M=F (p=0.934)	6.9±6.0 (0 – 20)	5.8±4.8 (0 – 17)	L=R (p=0.701)	-2.1±5.6 (-20 – 11)	-2.3±6.1 (-20 – 10)	-1.9±5.1 (-12 – 11)	M=F (p=0.988)	-1.0±7.0 (-20 – 11)	-3.2±3.5 (-9 – 0)	L=R (p=0.096)	-2.7±5.0 (-17 – 9)	-1.9±5.0 (-14 – 7)	-3.4±5.1 (-17 – 4)	M=F (p=0.405)	-2.4±5.9 (-17 – 7)	-2.9±4.1 (-12 – 4)	L=R (p=0.463)	-2.1±5.6 (-17 – 9)	-0.4±5.5 (-17 – 9)	-3.7±5.3 (-15 – 5)	M=F (p=0.05)	-2.4±5.5 (-15 – 5)	-1.7±5.7 (-17 – 9)	L=R (p=0.795)	-0.3±6.2 (-15 – 14)	1.4±6.6 (-12 – 14)	-1.9±5.6 (-15 – 5)	M=F (p=0.103)	-1.0±6.7 (-15 – 14)	0.5±5.8 (-12 – 12)	L=R (p=0.479)	PSP is 5 mm posteroinferior to SAL posterior end	6.6±4.2 (0 – 16)	7.3±4.2 (2 – 16)	6.0±4.1 (0 – 14)	.	.	No need for correction. Category B
21. SAL and F_L_–T_A_ intersection – inferior rolandic point	IRoP is at SAL and F_L_–T_A_ intersection	5.6±4.1 (0 – 15)	5.4±4.5 (0 – 15)	5.9±3.7 (0 – 12)	M=F (p=0.662)	5.5±4.1 (0 – 12)	5.8±4.1 (0 – 15)	L=R (p=0.818)	-1.3±3.7 (-12 – 5)	-2.4±4.5 (-12 – 4)	-0.3±2.3 (-7 – 5)	M=F (p=0.172)	-1.5±4.4 (-12 – 5)	-1.2±3.0 (-9 – 3)	L=R (p=0.899)	-2.3±4.5 (-10 – 11)	-0.6±5.0 (-9 – 11)	-3.9±3.3 (-10 – 2)	M≠F (p=0.017)*	-2.6±4.3 (-10 – 7)	-1.9±4.7 (-9 – 11)	L=R (p=0.622)	-1.8±5.5 (-12 – 15)	1.6±4.7 (-5 – 15)	-5.2±3.9 (-12 – 0)	M≠F (p<0.001)*	-2.2±4.7 (-11 – 10)	-1.5±6.3 (-12 – 15)	L=R (p=0.82)	-0.3±4.9 (-9 – 11)	2.9±4.1 (-5 – 11)	-3.5±3.2 (-9 – 2)	M≠F (p<0.001)*	-0.4±4.7 (-8 – 9)	-0.3±5.1 (-9 – 11)	L=R (p=0.931)	Male: IRoP is 5 mm anteroinferior to SAL and F_L_–T_A_ intersection. Female: IRoP is 5 mm posterior to SAL and F_L_–T_A_ intersection	4.1±2.9 (0 – 13)	4.6±3.3 (0 – 13)	3.7±2.5 (0 – 8)	.	.	No need for correction. Category B
22. O_M_ – C2 spinal process	O_M_ is projected at C2	10.5±7.5 (0 – 30)	11.9±6.4 (3 – 25)	9.0±8.3 (0 – 30)	M=F (p=0.067)	.	.	.	.	.	.	.	.	.	.	.	.	.	.	.	.	.	-2.0±12.9 (-25 – 30)	-6.5±12.1 (-25 – 11)	3.2±12.0 (-14 – 30)	M≠F (p=0.015)*	.	.	.	.	.	.	.	.	.	.	.	.	.	.	.	.	No need for correction. Category C
23. O_M_ – inion	Inion is at O_M_	57.4±12.9 (37 – 90)	53.7±8.6 (40 – 65)	61.0±15.4 (37 – 90)	M=F (p=0.072)	.	.	.	.	.	.	.	.	.	.	.	.	.	.	.	.	.	57.4±12.9 (37 – 90)	53.7±8.6 (40 – 65)	57.4±12.9 (37 – 90)	M=F (p=0.072)	.	.	.	.	.	.	.	.	.	.	Inion is 5.5 cm superior to O_M_	10.7±7.4 (3 – 35)	7.7±3.6 (3 – 15)	13.6±9.0 (5 – 35)	.	.	Accept correction. Category C
24. O_M_ – lambda	Lambda is at O_M_	128.0±13.4 (102 – 152)	125.0±9.7 (106 – 138)	132.0±15.6 (102 – 152)	M=F (p=0.065)	.	.	.	.	.	.	.	.	.	.	.	.	.	.	.	.	.	128.0±13.4 (102 – 152)	125.0±9.7 (106 – 138)	132.0±15.6 (102 – 152)	M=F (p=0.065)	.	.	.	.	.	.	.	.	.	.	Lambda is 13 cm superior to O_M_	10.5±8.3 (0 – 28)	8.1±7.6 (0 – 24)	12.9±8.5 (1 – 28)	.	.	Accept correction. Category C
25. O_M_ – calcarine sulcus	CaS is at O_M_	85.9±17.1 (60 – 120)	79.8±14.2 (60 – 104)	92.0±17.9 (69 – 120)	M=F (p=0.05)	.	.	.	.	.	.	.	.	.	.	.	.	.	.	.	.	.	85.9±17.1 (60 – 120)	79.8±14.2 (60 – 104)	92.0±17.9 (69 – 120)	M=F (p=0.05)	.	.	.	.	.	.	.	.	.	.	CaS is 8.5 cm superior to O_M_	14.8±8.3 (1 – 35)	12.2±8.6 (1 – 25)	17.4±7.3 (8 – 35)	.	.	Accept correction. Category D
26. O_M_ – parieto-occipital sulcus	POS is at O_M_	138.4±16.3 (113 – 178)	133.7±14.3 (116 – 155)	143.2±17.2 (113 – 178)	M=F (p=0.065)	.	.	.	.	.	.	.	.	.	.	.	.	.	.	.	.	.	138.4±16.3 (113 – 178)	133.7±14.3 (116 – 155)	143.2±17.2 (113 – 178)	M=F (p=0.065)	.	.	.	.	.	.	.	.	.	.	POS is 14 cm superior to O_M_	13.6±9.0 (1 – 38)	13.7±7.0 (3 – 24)	13.4±10.8 (1 – 38)	.	.	Accept correction. Category D
